# Recent Advances of Trained immunity in Macrophages

**DOI:** 10.7150/ijbs.115515

**Published:** 2025-08-11

**Authors:** Peizhi Li, Manman Liu, Yakun Liu, Jing Guo, Jingfeng Jiang, Guidan Wang, Chenlong Cao, Xinru Wang, Junxing Qu, Zhiheng Sun

**Affiliations:** 1 Department of Anesthesiology, Xinxiang First People's Hospital, The Affiliated People's Hospital of Henan Medical University, Xinxiang, Henan, China.; 2 Department of Intensive Care Unit, Xinxiang First People's Hospital, The Affiliated People's Hospital of Henan Medical University, Xinxiang, Henan, China.; 3 Department of Cardiology, Xinxiang First People's Hospital, The Affiliated People's Hospital of Henan Medical University, Xinxiang, Henan, China.; 4 Department of Pulmonary and Critical Care Medicine, Xinxiang First People's Hospital, The Affiliated People's Hospital of Henan Medical University, Xinxiang, Henan, China.; 5 College of Life Science, Institute of Biomedical Science, Henan Normal University, Xinxiang, Henan, China.; 6 State Key Laboratory of Cell Differentiation and Regulation, Xinxiang, Henan, China.; 7 Institutes of Health Central Plains, Henan Medical University, Xinxiang, Henan, China.; 8 Xinxiang Key Laboratory for Tumor Drug Screening and Targeted Therapy, Xinxiang, Henan, China.

**Keywords:** TI, Macrophage, Cancer, Infectious disease, Autoimmune diseases.

## Abstract

Trained immunity (TI), also known as innate immune memory, is the long-term change in the functional program of innate immune cells after transient stimulation through epigenetic and metabolic alterations. This reprogramming augments the response to secondary challenges fueled by various stimulus and contributes to the resistance of cancer, infectious diseases, auto-inflammatory disorders, and other diseases. Macrophages, which are versatile innate immune cells with remarkable plasticity, can adapt to different microenvironments and perform diverse functions. TI of macrophages has been deeply involved in pathogen infection. However, current understandings in the effect of TI are still incomplete and require further investigation and summarization. In this review, we summarized the existing knowledge in this field including the hallmark and mechanism of TI, its impact on health and disease, as well as the potential as a therapeutic tool. This study provides new perspectives for a comprehensive insight of TI.

## Introduction

TI describes the process by which innate immune cells acquire immunological memory. It is induced upon exposure to exogenous or endogenous insults and then returns to an inactive state. Subsequent exposure to a second specific or non-specific stimulus leads to enhanced effector functions. Trained cells can leverage the general memory of inflammation to counter entirely new threats 1. TI is based on two main pillars: epigenetic and metabolic reprogramming. In contrast to classical innate immunity, TI is coordinated through epigenetic reprogramming, defined as sustained changes in transcription programs via epigenetic rewiring, leading to changes in cell physiology that do not involve permanent genetic changes such as mutations and recombination 2. TI reflects the property of the innate immune system to mount a memory-like response to past microbial and non-microbial challenges. To date, research has demonstrated that TI can be sustained in innate immune cells for periods ranging from months to decades. In bone marrow progenitor cells, it is termed central TI, while in circulating mononuclear macrophages, it is referred to as peripheral TI. Given that myeloid progenitor cells have a limited lifespan, the maintained TI in these cells does not endure as long as that of peripheral TI 3.

In the realm of inflammation research, innate immune cells within a TI milieu can identify foreign bacterial cell wall constituents, viral products, and bacterial nuclear components via pattern recognition receptors (PRRs) expressed on the cell surface and within the cytoplasm. These recognized biochemical motifs are termed as pathogen-associated molecular patterns (PAMPs) 4. Significantly, this immune defense mechanism can also be activated by the release of damage - associated molecular patterns (DAMPs). DAMPs are generated by immune cells that have been activated in a sterile inflammatory setting due to damaged or necrotic tissue 5. PAMPs and DAMPs induce the activation of PRRs in innate immune cells, a process that promptly elicits the secretion of pro - inflammatory cytokines, such as interleukin - 6 (IL-6), interleukin - 1β (IL-1β), and tumor necrosis factor-α (TNF-α) 6, 7. In a strikingly different mechanism to TI, endotoxin tolerance (ET) presents with a transiently attenuated state of innate immune cells during subsequent challenges. This phenomenon serves to safeguard the body by preventing the immune system from inflicting excessive physical damage while still maintaining its ability to eliminate pathogens. The key manifestations of ET include a reduction in pro-inflammatory features and an upregulation of anti-inflammatory patterns 8. Both the trained immune response and the tolerance response primarily stem from alterations in diverse types of PRRs, including Toll-like receptors (TLRs), nucleotide-oligomerization domain (NOD)-like receptors (NLR), retinoic acid-inducible gene I (RIG-I)-like receptors (RLRs), and C-type lectin receptors (CLRs). These receptors play a crucial regulatory role in local and systemic inflammatory responses within organisms 9. Overall, the activation of these signaling pathways leads to TI and immune tolerance, predisposing to the development of inflammatory diseases.

Macrophages serve as the vanguard in the defense against infections and are an indispensable component of innate immunity 10. Initial insights into the adaptive properties of macrophages were gleaned from investigations on lipopolysaccharide (LPS)-induced tolerance 11. Subsequently, it was revealed that upon secondary stimulation with Candida albicans, macrophages demonstrated enhanced production of pro-inflammatory cytokines, essentially manifesting a TI phenotype. Macrophages express a diverse and unparalleled array of innate immune receptors, such as Toll-like receptors (TLRs), inflammasomes, and lectin-like receptors, which are strategically positioned in the cell membrane, cytoplasm, and endosomal compartments 12. In recent years, research has underscored the substantial contributions of macrophages to host defense during the process of TI. Their ability to recognize and respond to a wide range of pathogens, combined with their capacity for immunomodulation, renders them pivotal players in the complex landscape of the immune response.

With the in-depth exploration of the mechanism of macrophages in TI, it is expected to provide crucial targets for the development of novel immunomodulatory therapies, facilitating the conquest of numerous inflammatory diseases. Meanwhile, continuous exploration of the complex signaling pathways involved in TI and the synergistic interactions among various immune cells will expand the boundaries of our understanding of the immune system and open broader prospects for precision medicine. In this review, we summarize the polarization of macrophages and their potential roles in TI against diseases and provided an outlook on the development of TI for its future application in disease treatment.

## Basic Concepts of TI

### Differences from conventional innate immunity

Innate immunity serves as the body's first line of defense against invading pathogens, a system that all animals possess from birth. Innate immune memory, namely TI, differs from the traditional innate immunity 13. Firstly, TI challenges the traditional view that only adaptive immunity can establish memory immunity. It indicates that the innate immune system, when subjected to the same or different re-stimulation, is capable of promptly generating a non-specific memory function against other pathogens 14. In contrast, traditional innate immunity is considered to lack memory. When the antigen initially stimulates the immune system, the cells do not differentiate into memory cells, and upon re-exposure to the antigen, they cannot rapidly mount a strong immune response. Secondly, although TI is non-specific, it can produce a faster and stronger immune response to re-stimulation, regardless of whether the stimuli are the same or different. The innate immune response lacks specific selectivity and is not specific to a particular antigen 15. Thirdly, the immunological phenotype of TI has been proven to last for at least three months and even up to one year 16. Although shorter than the memory of adaptive immunity, it represents the long-term adaptation of innate immune cells. Traditional innate immune responses are typically transient and do not involve long-term immune memory. Moreover, TI is induced by epigenetic reprogramming of signaling pathways rather than gene rearrangement 17. However, traditional innate immunity does not involve long-term epigenetic reprogramming, and its responses are rapid but not persistent. Finally, the cells capable of being induced to generate TI include monocytes, macrophages, dendritic cells, neutrophils, natural killer cells, innate-like lymphocytes, and hematopoietic stem cells 18. The cells involved in traditional innate immunity mainly comprise macrophages, neutrophils, etc., which are primarily responsible for non-specific phagocytosis of foreign pathogens 19. In summary, the main differences between TI and traditional innate immunity lie in memory function, specificity, duration, mechanism, and cell types. TI provides the innate immune system with a memory function like that of adaptive immunity, enhancing the body's defense against re-infection.

### Non-specific response

TI does not target specific pathogens; instead, it augments the capacity of innate immune cells to react to stimuli derived from diverse pathogenic sources. Studies have indicated the existence of adaptive traits within an innate immunity system. For instance, treatment with a non-proteinaceous vaccine composed of aluminum hydroxide, monophosphoryl lipid A, and fungal mannans can improve the survival rate of mice and reduce the pathogen load of invasive bloodstream or pulmonary infections caused by *methicillin-resistant Staphylococcus aureus*, *vancomycin-resistant Enterococcus faecalis*, *extended-spectrum β-lactamase-producing Escherichia coli*, *carbapenem-resistant Acinetobacter baumannii*, *Klebsiella pneumoniae*, and *Pseudomonas aeruginosa*. It can also prevent infections by the *fungi Rhizopus Delemar* and *Candida albicans* 20. Additionally, studies showed that mice transplanted with influenza-cultured hematopoietic stem and progenitor cells (HSPCs) exhibit enhanced immunity against *Mycobacterium avium* attacks and vice versa, demonstrating a cross-protective effect against antigenically distinct pathogens 21. These data confirm that either the infection itself or the exposure of the immune system to immunostimulants can trigger broad-spectrum defense against subsequent assaults by the same or other pathogens. This non-specific protection argues against the dominant role of the adaptive immune system in mediating the type of cross-protection and instead activates non-specific, protective innate immune mechanisms.

### Memory response

It has been believed that immune memory is the unique hallmark of adaptive immune responses. However, increasing studies have suggested that innate immune cells can exhibit adaptive characteristics, which challenge this dogma 22. Certain stimuli can induce alterations in DNA methylation patterns (such as hypomethylation of CpG islands), stably maintaining transcriptional programs that persist for weeks to months. Plants and invertebrates lack the acquired immune system, but they have also been reported to possess a stronger ability to resist reinfection 23. Research has demonstrated that copepods depend only on the innate immune system. Intriguingly, they possess an immune memory that can prevent reinfection by parasitic tapeworms 24. Subsequently, the existence of innate immune memory has been identified in various other invertebrate and mollusks species. This phenomenon is highly conserved and widespread throughout the evolutionary of innate immune memory across species 25. TI is regulated by distinct mechanisms and exhibits lower specificity compared to adaptive immune memory. Nevertheless, both TI and adaptive immune memory fulfill the same fundamental function: enabling a more efficient and robust response to pathogens, thereby enhancing the host's survival rate.

### Multiple stimuli

In the realm of immunology, TI, as an emerging and pivotal immune mechanism, is progressively shattering traditional understandings. It endows the body's immune system with a non-specific “memory” capacity, enabling it to respond promptly and robustly when confronted with pathogen incursions. The activation of this distinctive immune function is inextricably linked to the induction by a series of specific substances. Exploration of the inducers of TI not only facilitates our comprehension of immune regulation but also blazes a trail for the prevention and treatment of numerous diseases. Subsequently, a detailed exposition of several proven induces of TI will be presented.

#### β-Glucan

β- Glucan stands as an efficacious inducer in innate immune cells and is among the most extensively utilized model stimuli in the study of TI 26. Mechanistically, β-glucan is recognized by the transmembrane CLR (Dectin-1), which subsequently initiates intricate intracellular train events 27. The trained properties of β-glucan were initially demonstrated through peritoneal injection in mice *in vivo*. This experimental treatment augmented myelogenesis and elicited an effective response upon subsequent re-stimulation. Notably, the trained mice were shielded from chemotherapy-induced myelosuppression, highlighting the potential of β-glucan in modulating the immune response in the backdrop of myeloid cell production and protection against chemotherapy-related hematopoietic toxicity 28. An investigation revealed that the transition of cell metabolism from oxidative phosphorylation to glycolysis, mediated by the AKT-mTOR-HIF1α signaling pathway, constitutes the crucial metabolic foundation for β-glucan-induced TI in monocytes 29. In subsequent investigations, a comprehensive integrative analysis of transcriptomics and metabolomics further elucidated that glycolysis, glutaminolysis, and cholesterol biosynthesis are the three major non-overlapping metabolic pathways essential for β-glucan-induced macrophage training. When any one of these pathways is impeded, it restricts the induction of TI 30. Experiments have confirmed that β-glucan can epigenetically restructure monocytes, giving rise to distinctive IL-1 signaling 7, 31. Moreover, β-glucan pretreatment mitigated the histopathological alterations inflicted viral-induced on the mouse lung and enhanced the expression of IFN-β 32. And, β-glucan-induced training immunophenotype of macrophages can inhibit tumor growth through the targeted delivery of nanoparticles, which acts synergistically with immune checkpoint inhibitors 33. The therapeutic efficacy of TI also faces core challenges in clinical translation. For example, the enhancing effect of TI exhibits a strict dose window: low-dose β-glucan can induce protective TI via the glycolysis-epigenetic axis, whereas high doses may trigger excessive inflammation or even cytokine storms. Moreover, there are substantial inter-patient differences in the basal states of immune cells; for instance, monocytes from diabetic patients show disordered metabolic reprogramming due to long-term hyperglycemic environments, leading to reduced efficiency in TI induction. Developing personalized dosing regimens based on individual immune profiles remains a critical hurdle in clinical implementation.

#### Bacillus Calmette-Guérin (BCG)

BCG is an attenuated strain of *Mycobacterium Bovis* obtained via continuous passage 34. BCG induces TI by binding to NOD receptors 35. BCG vaccination induces histone modification of macrophages, upregulating the expression of cell surface molecules such as CD14, NOD2, CD11b, and mannose receptor. Upon reinfection, it can rapidly activate downstream signaling pathways, promoting the release of diverse cytokines, including IL-1β, IL-6, TNF-α, IFN-γ, and IL-6 2. The initial application of BCG was for the prevention of tuberculosis through intradermal injections 36, and subsequent developments and applications have demonstrated that the protective effect of BCG can be independent of T/B cells 37. The TI induced by BCG bears a high degree of resemblance to that induced by β-glucan 38. Nevertheless, a crucial distinction exists between the TI-induced by BCG and β-glucan. During BCG-induced, both glycolysis and oxidative phosphorylation are upregulated. As a result, the characteristic Warburg effect, which is commonly seen in macrophages induced of β-glucan, is not observed 39. The glycolysis, glutamine metabolism, and cholesterol synthesis mediated by the mTOR signaling pathway, which are essential for β-glucan trained, are also indispensable for BCG-induced TI 40. The IL-1 pathway also plays a pivotal role in BCG-induced-macrophages. Intriguingly, the induction of BCG-mediated trained macrophages necessitates IFN-γ rather than IL-1 signaling pathway in mice 3. Moreover, clinical trials have confirmed that unlike the MMR vaccine, which relies on γδ T cells to induce TI, the protective effect of BCG is primarily mediated by epigenetic reprogramming of macrophages, highlighting the cell-type specificity of TI induction across different vaccines 41, 42. Experimental evidence has also shown that varying doses of BCG injection exhibit no significant differences-standard dosing is sufficient to induce TI in monocytes, obviating the need for revaccination or high-dose administration. This finding provides a basis for optimizing vaccination protocols in resource-limited regions. Additionally, individuals with baseline immunocompromise (such as neonates and the elderly) may derive stronger heterologous protection from BCG, as their lower basal cytokine levels allow for greater inductive capacity of TI 43. Research has revealed that BCG can induce non-specific protective effects against other pathogens through the process of TI. In addition to its capacity to prevent tuberculosis as previously stated 44, BCG can also combat cancers such as bladder cancer 45 and melanoma 46. Moreover, early-life BCG vaccination demonstrates inverse correlations a risk reduction of lung cancer and leukemia 47, 48.

#### LPS

Lipopolysaccharide (LPS), found in the outer membrane of Gram-negative bacteria, executes diverse functions in biology. Prior investigations have illustrated the suppression of essential immunosuppressive mediators, such as PI3K and IRAK-M, accompanied by IRAK-1 and Tollip. These processes act as potential mechanisms underpinning the commencement of LPS-induced macrophage activation, giving rise to a protracted and moderate pro-inflammatory state 49. This functional reprogramming (tolerance) of macrophages is attributed to the transcriptional silencing of the majority of genes induced by LPS. When tolerant macrophages are restimulated with LPS, transcriptional silencing occurs concomitantly with an inability to mark histones on the promoters of tolerant genes 50, 51. Notably, clinically ultra-low-dose LPS has been found to induce perennial innate immune activation through processes associated with TI, despite both being mediated by cell-surface TLR4.The increased hematopoiesis-related processes associated with TI exhibit beneficial aspects following LPS restimulation. For instance, it protects mice from chemotherapy-induced myelosuppression 28. Individuals infected with the human immunodeficiency virus show increased levels of IL-1β production following LPS and bacterial infections 52. Data indicates that LPS-trained changes in hematopoietic stem cells can significantly improve the response to secondary bacterial infections, such as *Pseudomonas aeruginosa* 53. Meanwhile, the efflux of cellular cholesterol in macrophages triggered by ultra-low-dose LPS is suppressed through downregulating the formation of lipid transporter proteins SR-B1, ABCA1 and ABCG 54. However, the role of cholesterol metabolism in LPS-induced TI remains to be determined.

#### Other infectious agents and related substances

Apart from BCG and β-glucan, other infectious agents also serve as natural inducers of TI. Respiratory tract infections caused by adenovirus can endow alveolar macrophages with lasting memory, which is maintained by an enhanced TI phenotype at local mucosal sites 55. This may lead to a robust defense of the host against heterologous bacterial infections. Trained macrophages require the assistance of CD8^+^T cells during the initiation phase but subsequently self-sustain in the alveoli and become independent of circulating monocytes. They exhibit characteristics of TI, such as a defense-ready profile and a high glycolytic rate. Recent studies have demonstrated that hormones can induce TI, particularly sex-related hormones(estradiol) or aldosterone that interprets homeostasis and influences numerous cardiovascular inflammatory events 56, 57. Saturated fat or the secretome of obesity origin obtained from obese mice induces cells to synergistic activation of glycolysis and mitochondrial respiration and boost endotoxin-induced production of TNFα and IL-6 58. Consequently, this stimulates the development of innate immune memory. Macrophages deficient in insulin signaling display diminished responses to pathogens and modified metabolism, indicating that insulin resistance embodies a state of TI 59. Du et al. discovered that hypoxia-preconditioned stromal progenitor cells can secrete paracrine factor-insulin-like growth factor (IGF)-2, which trains maturing murine macrophages towards an M2-like phenotype, thus suppressing autoimmune inflammation 60. Moreover, monocytes are isolated from patients with pheochromocytoma or paraganglioma may be significantly affected in terms of their function, transcription, and epigenetics following long-term exposure to high catecholamine levels 61. Fatty acid synthesis is a crucial pathway for inducing TI, and pharmacological inhibition of this pathway can attenuate aldosterone-induced TI 56. Persistent hypercholesterolemia induces a TI phenotype in monocytes, increasing an individual's risk of developing cardiovascular diseases, and this pro-inflammatory phenotype cannot be reversed even by statins 62. In addition, oxidized low-density lipoprotein (oxLDL) also induces TI through the glycolytic pathway 63. Oroxylin A (OA) induces TI by targeting the MIF-IL-6 axis and modulating immune cell subsets, offering a novel molecular target for the prevention and treatment of sepsis-associated cancers 64. Certain inducers can initiate TI independently of metabolic pathways; for example, resveratrol, a natural polyphenol, primarily triggers TI through modifications on the IL-6 and TNF-α promoters without enhancing-or even inhibiting-glycolysis, oxidative phosphorylation, and reactive oxygen species production 65.

In summary, TI is an essential element of the body's immune defense system. The diversity and complexity of its inducers offer extensive opportunities for further investigation into immune regulatory mechanisms. These inducers range from classic factors such as β-glucan and BCG vaccine, to LPS with dual characteristics, to adenovirus infection, hormones, and other stimuli. Each of these inducer's shapes TI via distinct pathways. Current models are predominantly based on *in vitro* cell cultures or single stimuli, lacking simulations of *in vivo* multi-pathogen and multi-stimulation scenarios. Integration of technologies such as single cell sequencing and *in vivo* imaging is necessary to dissect this heterogeneity.

### Metabolic-epigenetic crosstalk in TI in diseases

The formation of immunological memory in TI relies on the synergistic interplay between metabolic reprogramming and epigenetic modifications 66. Metabolic intermediates, serving as substrates for epigenetic regulation, reshape chromatin states via histone modifications and DNA methylation. These two processes establish a dynamic signaling network to enable long-term alterations in gene expression, thereby sustaining the long-lasting memory phenotype of macrophages.

#### Cell metabolism and epigenetics

**Figure 1:**
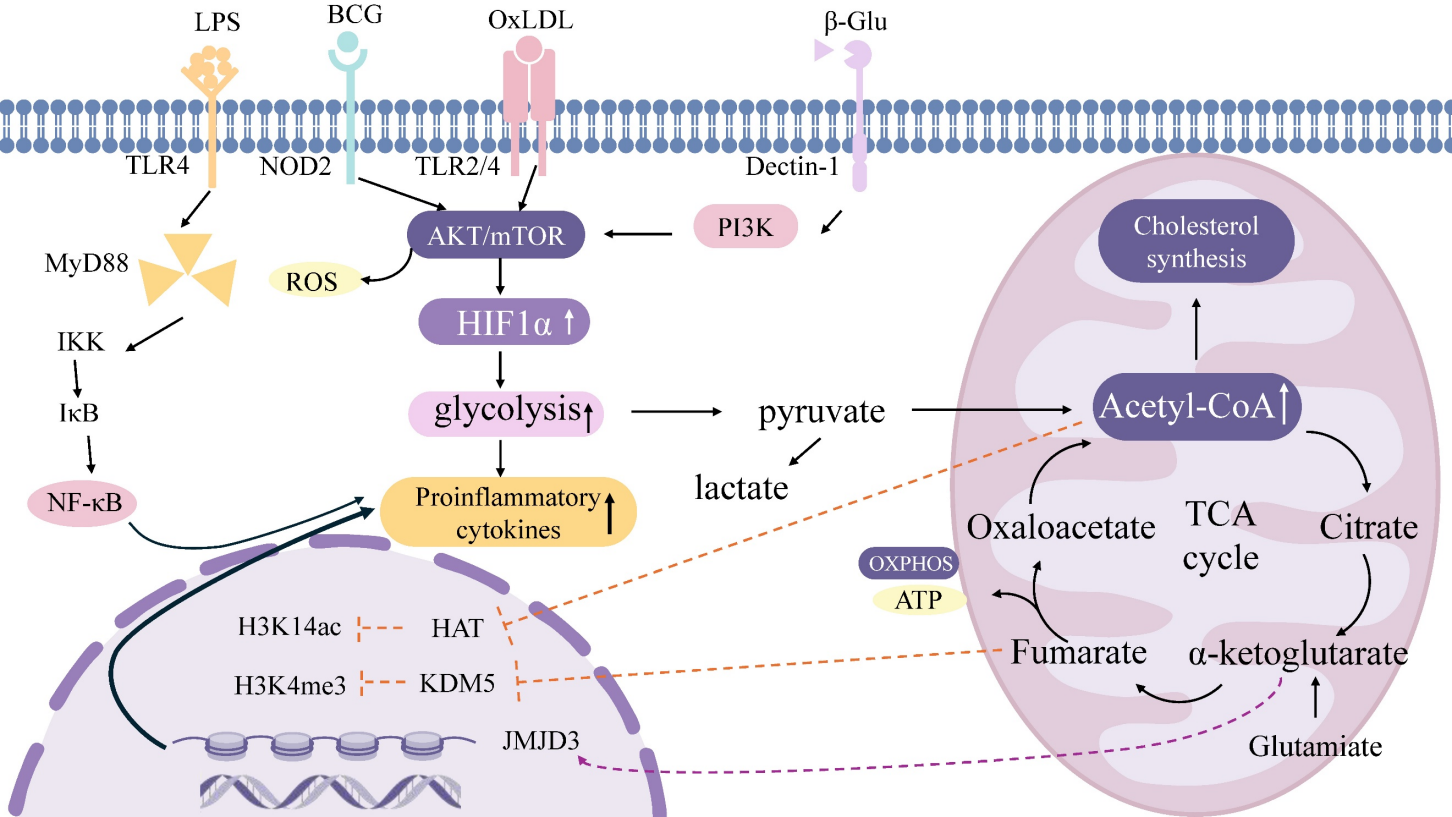
TI-related signaling pathways and metabolic processes. Immune inducers such as β-glucan, BCG, LPS, and oxLDL can enter the cytoplasm through different receptors and then activate the Akt-mTOR-HIF1α axis to initiate a series of intracellular cascades that trigger upregulation of various metabolic pathways. These pathways include glycolysis, tricarboxylic acid cycling, OXPHOS, and glutamine decomposition. Metabolites obtained from these pathways, such as fumarate and acetyl-CoA (acetyl-CoA), can in turn mediate epigenetic remodeling of histones; This remodeling left behind epigenetic marks such as H3K4me3, H3K9me3, and H3K27ac. Acetyl-coa also regulates the cholesterol synthesis pathway through mevalonate. BCG, Bacille Calmette-Guérin; LPS, Lipopolysaccharides; oxLDL, oxidized low-density lipoprotein; mTOR, mammalian target for rapamycin; OXPHOS, oxidative phosphorylation; PI3K, Phosphoinositide 3-Kinase; NF-κB, nuclear factor kappa-B; ROS, Reactive oxygen species; ATP, adenosine triphosphate; TCA, tricarboxylic acid cycle.

Metabolism establishes the material foundation for epigenetic regulation 67. Acetyl-CoA generated via glycolysis acts as a substrate for histone acetyltransferases (HATs), facilitating activating modifications such as H3K14ac. For example, β-glucan drives glycolysis through the Akt-mTOR-HIF1α axis, elevating acetyl-CoA production and subsequently augmenting H3K4me3 and H3K27ac modifications at the promoters of pro-inflammatory genes 29. Additionally, impaired glutathione synthesis in β-glucan-trained macrophages reduces the levels of the methyltransferase enhancer of zeste homolog 2 (EZH2), thereby inhibiting EZH2-mediated amplification of heterologous immune protection 68. The glucose metabolite fumarate sustains epigenetic memory by inhibiting histone demethylases (KDM5) 30. During Candida infection, α-ketoglutarate (α-KG) promotes M2 macrophage polarization through JMJD3 activation 69. TI induced by the BCG vaccine concurrently activates glycolysis and OXPHOS, with ATP generated from mitochondrial respiration supporting the activity of epigenetic modifiers. Post-BCG stimulation, H3K4me3 accumulates at the promoters of receptor genes such as CD14 and NOD2 in macrophages, enhancing pathogen recognition 70. OxLDL upregulates cholesterol synthesis via OXPHOS, and its metabolite mevalonate contributes to H3K4 methylation, driving monocyte polarization toward a pro-inflammatory phenotype 71, 72. Epigenetic mechanisms dynamically regulate metabolic networks 73. Enrichment of H3K4me3 at the promoters of key pentose phosphate pathway enzymes (e.g., G6PD) suppresses this pathway and enhances glycolytic flux 74. The β-glucan-induced lncRNA UMLILO maintains the expression of glycolysis-related genes by recruiting H3K4me3 modifications, enhancing antibacterial memory in monocytes 75. H3K27me3 represses the transcription of OXPHOS-related genes (e.g., SDHA, CYCS) 76 (Figure 1).

#### Cancer

In Hepatocellular carcinoma (HCC), BCG-induced glycolysis enhances M1 macrophage infiltration, synergizing with IFN-γ to inhibit tumor growth 77. In tumor-associated macrophages (TAMs), OXPHOS and fatty acid oxidation dominate, mediating H3K4me3 modifications via PPARγ to promote anti-inflammatory genes (e.g., ARG1, MRC1) and drive tumor progression 78. In ovarian cancer, circITGB6 stabilizes pro-tumor transcripts by binding to IGF2BP2/FGF9 mRNA, promoting H3K4me3 enrichment at M2 polarization gene promoters to enhance cisplatin resistance 79. BCG vaccine enriches H3K4me3 modifications at innate immune receptor gene promoters in macrophages, augmenting their antigen-presenting capacity and enhancing immune responses in bladder cancer 80. Clinical trials have also confirmed that TI induced by β-glucan can inhibit the proliferation of pancreatic cancer cells 81.

#### Infectious diseases

Influenza virus-induced alveolar macrophages maintain OXPHOS via fatty acid oxidation, with PPARγ-mediated H3K4me3 modifications suppressing glycolysis to avoid tissue damage from lactate accumulation. Meanwhile, H3K4me3 modifications at the IFN-β gene promoter persist for 4 months, providing long-term antiviral protection 82. In BCG-induced macrophages, α-ketoglutarate derived from glutamine catabolism promotes H3K4me3 modifications at M2 polarization-related genes (e.g., ARG1) via the JMJD3 histone demethylase, enhancing mycobacterial clearance capacity 83. V132 drives metabolic rewiring to increase glycolysis and oxidative phosphorylation, induces elevated H3K27 acetylation (H3K27ac), and reduces the repressive mark H3K9 trimethylation (H3K9me3), thereby enhancing transcription of pro-inflammatory genes IL6 and TNF-α 84. β-glucan activates the Akt-mTOR-HIF1α axis via the Dectin-1 receptor, where glycolytic intermediate acetyl-CoA drives H3K14ac modifications to enhance IL-17 secretion during Candida infection, while conferring cross-protection against methicillin-resistant Staphylococcus aureus (MRSA) infection via epigenetic memory 85.

#### Autoimmune diseases

In synovial cells of rheumatoid arthritis (RA) patients, a high-glucose environment sustains NF-κB pathway activation via glycolysis-dependent H3K4me3 modifications, promoting secretion of IL-6 and TNF-α 86. Dysregulated OXPHOS increases mitochondrial ROS production, further reinforcing histone methylation to form an inflammatory positive feedback loop. In a palmitic acid-induced cell model of gestational diabetes mellitus (GDM), mitochondrial dysfunction and ROS generation reduce H3K4me3 enrichment at pro-inflammatory cytokine loci, augmenting their production to maintain normal pregnancy 87. In myeloid cells of systemic lupus erythematosus (SLE) patients, type I interferons (IFN-I) induce H3K4me3 enrichment at promoters of IFN-stimulated genes (ISGs) via the JAK-STAT pathway 88. In the mouse model of systemic lupus erythematosus, metabolic alterations in glycolysis and oxidative phosphorylation (OXPHOS) within kidney-derived macrophages are closely associated with the production of inflammatory cytokines and chemokines by these cells. Administration of the glycolytic inhibitor 2-deoxy-D-glucose (2-DG) has been shown to reduce the expression levels of IL-6, TNF-α, and IL-1β in renal tissue 89.

#### Atherosclerosis

OxLDL activates glycolysis via the NLRP3 inflammasome, with increased mitochondrial reactive oxygen species (ROS) inducing oxidative inactivation of KDM6A. This elevates H3K27me3 levels, suppresses the expression of cholesterol reverse transport genes (e.g., ABCA1), and promotes foam cell formation 70. In diabetic patients, hyperglycemia enhances the binding of the Runx1 transcription factor to pro-inflammatory genes via glycolysis-dependent H3K4me3 modifications, driving atherosclerosis progression 90. Knockdown of KMT5A reduces H3K4me3 modifications and inhibits SYK kinase activity, decreasing atherosclerotic plaque area in a myocardial infarction model. Additionally, gut microbiota dysbiosis triggers glycolipid metabolic disorders, elevating circulating LPS and TMAO levels to induce TI, ultimately promoting atherosclerotic formation 91. Phenotypic changes in macrophages following TI, such as increased glycolysis, indicate that a high-glucose environment exacerbates the pro-inflammatory consequences of TI, accelerating atherosclerotic progression in diabetic patients. Specifically, in the context of β-glucan-mediated macrophage training, genome-wide analysis of H3K4me3 reveals epigenetic activation of numerous genes involved in atherosclerotic pathogenesis. These genes encode not only pro-atherosclerotic cytokines and chemokines but also proteins associated with foam cell formation and plaque vulnerability 92, further implying that mechanisms driving TI possess pro-atherosclerotic effects, potentially contributing to ASCVD (atherosclerotic cardiovascular disease) development. The current understanding of TI primarily derives from *in vitro* experiments or animal models, necessitating caution when translating these findings into clinical therapy for ASCVD patients. For instance, monocytes from symptomatic atherosclerotic patients exhibit a TI phenotype with epigenetic rewiring characterized by reduced histone mark H3K4me3 at pro-inflammatory genes-distinct from the H3K4me3 elevation observed *in vitro* TI models 93. Upon entering different tissues, macrophages generate a new metabolic signature-metabolic reprogramming-to adapt to tissue microenvironments, commonly involving fatty acid synthesis, lactate metabolism, cholesterol metabolism, etc. 94, 95.

The metabolic and epigenetic mechanisms of TI (TI) together constitute the molecular basis of innate immune memory. Metabolic reprogramming provides substrates (e.g., acetyl-CoA, α-KG) for epigenetic modifications, while epigenetic marks (e.g., H3K4me3, H3K27ac) maintain metabolic memory by regulating gene expression profiles. The crosstalk between these two systems exhibits disease-specific regulatory features. In pathological contexts, this network can either exert protective effects by enhancing anti-bacterial and anti-tumor immunity or exacerbate chronic inflammation through metabolic-epigenetic dyshomeostasis.

**Figure 2:**
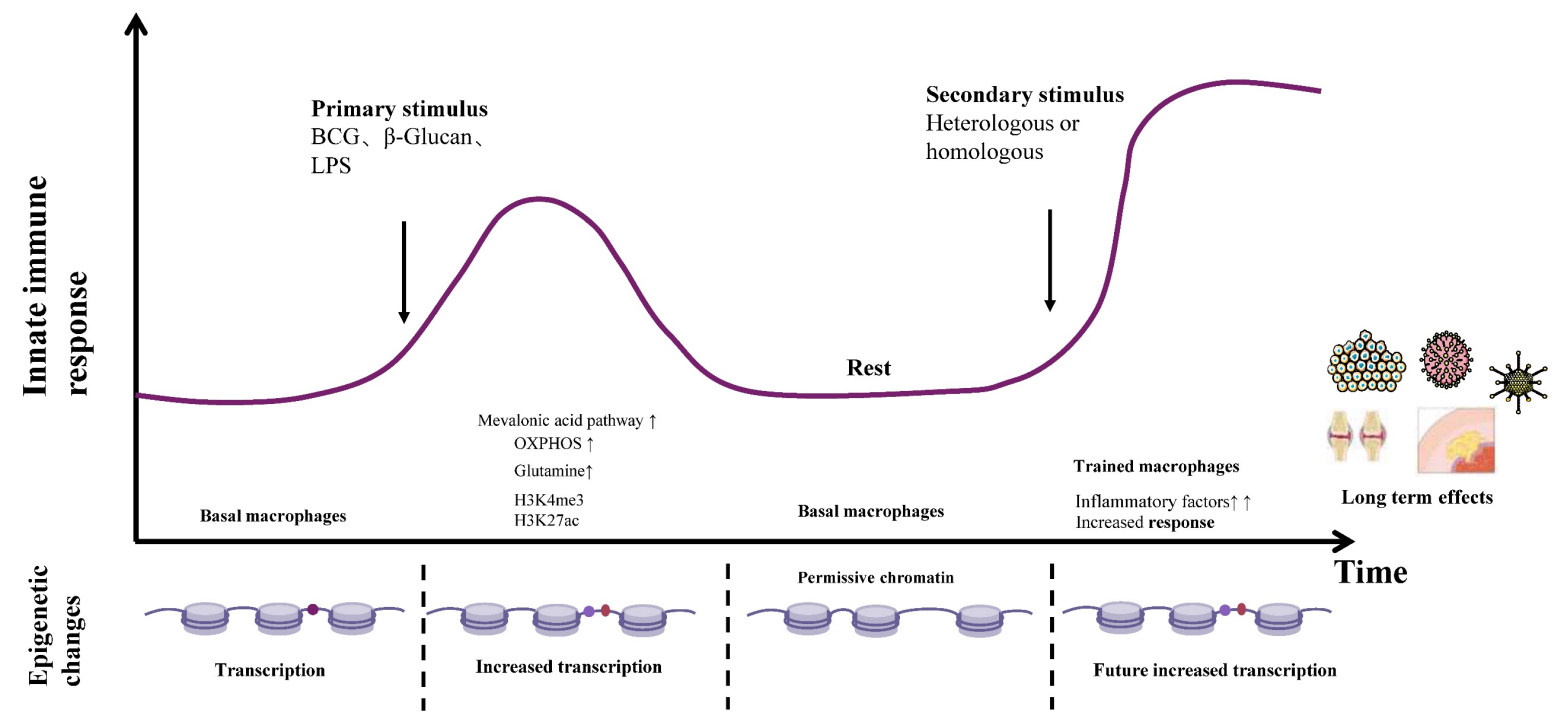
The formation and maintenance of immune memory for TI. Schematic depicting the quantitative alterations and dynamic shifts in the epigenetic landscape corresponding to distinct stages of macrophage training, alongside their consequential disease impacts. Macrophages across tissues establish an inflammatory memory state - characterized by transcriptionally inactive yet permissive chromatin - enabling the rapid recruitment of signal-dependent transcription factors and enhanced transcription of target genes upon heterologous or homologous secondary challenge. The top panel illustrates diverse stimuli (including BCG, LPS, and oxLDL) that engage cognate surface receptors, instigating profound epigenetic reprogramming and metabolic pathway rewiring within tissue-resident macrophages. This process entails elevated histone methylation and acetylation, coupled with augmented oxidative phosphorylation, mevalonate pathway flux, cholesterol metabolism, and glutaminolysis. Consequently, these trained macrophages exhibit heightened inflammatory mediator production, thereby conferring anti-tumor effects and protection against infectious diseases, while conversely demonstrating dual-edged consequences in the context of autoimmune disorders and atherogenesis. β-Glu, β-glucan; BCG, Bacille Calmette-Guérin; LPS, Lipopolysaccharides; oxLDL, oxidized low-density lipoprotein; OXPHOS, oxidative phosphorylation.

Among numerous epigenetic alterations in diseases, identifying the most critical one that can serve as an activation/inhibition target remains to be fully elucidated. In TI-related metabolism, while glycolysis forms the metabolic foundation, other pathways such as lipid, protein, and nucleotide metabolism may also play therapeutic roles. The epigenetic imprints of TI (e.g., H3K4me3 modifications) can persist for months to years, but their prolonged existence may induce chronic inflammation or metabolic disorders. For example, in atherosclerosis, persistently activated TI promotes cholesterol deposition via the mevalonate pathway, accelerating plaque progression. Additionally, immunosenescence may impair TI induction efficiency, as evidenced by the significantly reduced protective efficacy of BCG vaccine in the elderly. Thus, the long-term safety and memory sustainability of TI-based disease therapies require careful consideration. Deciphering dynamic mechanisms via single-cell technologies, developing precise targeting tools, and optimizing delivery systems hold promise for translating TI from basic research into widely applicable therapeutic strategies, opening new avenues for preventing and treating infections, tumors, and immune diseases (Figure 2).

## Organ-Specific Macrophage TI

### Dual lineage origins and heterogeneity

Macrophages represent the first identified TI cells exhibiting adaptive immune characteristics, with such immune memory documented in infections by bacteria, fungi, and parasites. These cells exhibit high heterogeneity and can rapidly alter their functions in response to local microenvironmental signals 96. Macrophages originate from two distinct lineages: one derived from bone marrow hematopoietic stem cells (HSCs) that differentiate through monocytes, a process regulated by microenvironmental signals such as colony-stimulating factors (M-CSF, GM-CSF) and cytokines (IFN-γ, IL-4). This lineage progresses sequentially from HSCs to myeloid progenitors, then to monocytes, and finally matures into macrophages 97. The other lineage primarily originates from embryonic yolk sac or fetal liver progenitors, possesses self-renewal capacity, and is independent of circulating monocyte replenishment-exemplified by tissue-resident macrophages 98.

### Functional polarization

**Figure 3:**
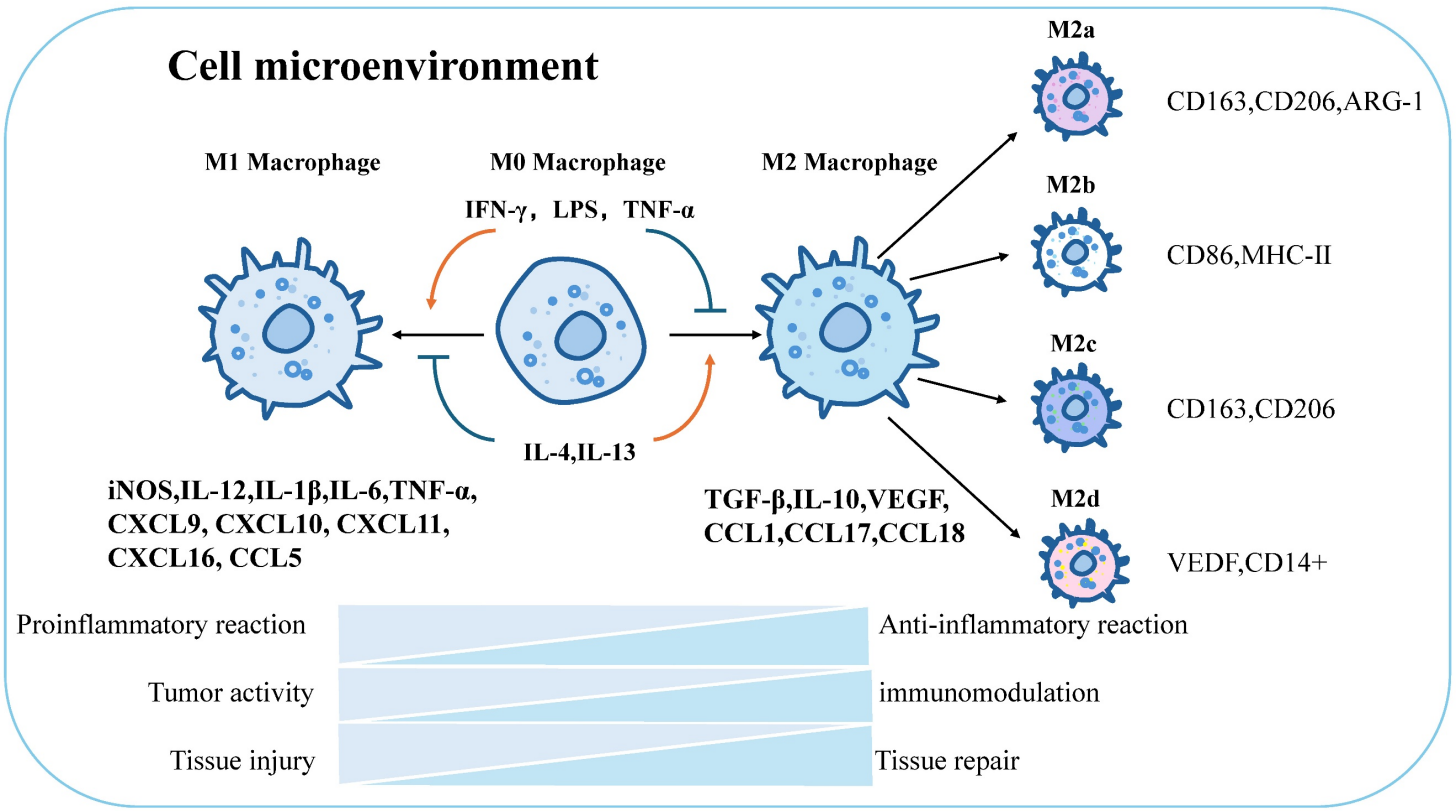
Global characterization of macrophage polarization and immune function. Macrophages will be polarized in response to different stimuli, and will be polarized toward M1 when stimulated by IFN-γ, LPS, TNF-α, and M2 when stimulated by IL-4,IL-13. M1 and M2 have different physiological functions. M1 is mainly responsible for proinflammatory effect, tumor activity and tissue damage, while M2 macrophages are mainly responsible for anti-inflammatory effect, immune regulation and tissue repair. In addition, M2 macrophages can continue to be polarized into four different types: M2a, M2b, M2c, and M2d. The respective surface markers are: M2a:CD163,CD206,ARG-1; M2b:CD86,MHC-Ⅱ; M2c :CD163,CD206; M2d:VEDF, CD14+. iNOS, inducible nitric oxide synthase; IL-12, interleukin12; IL-1β, interleukin1β; IL-6, interleukin6; TNF-α, tumor necrosis factor α; CXCL9, C-X-C Motif Chemokine Ligand 9; CXCL10, C-X-C Motif Chemokine Ligand 10; CXCL11, C-X-C Motif Chemokine Ligand 11; CXCL16, C-X-C Motif Chemokine Ligand 16; CCL5, C-C Motif Chemokine Ligand 5;TGF-β, Transforming growth factor-β; IL-10, interleukin10; VEGF, vascular endothelial growth factor; CCL1, C-C Motif Chemokine Ligand 1; CCL17, C-C Motif Chemokine Ligand 17; CCL18, C-C Motif Chemokine Ligand 18.

Macrophages can be defined and classified based on their functions (e.g., phagocytosis, immunity) and specific markers (e.g., CD80, CD163) 99. Stimulation with Th1 cytokines and granulocyte-macrophage colony-stimulating factors (GM-CSF) drives macrophage polarization toward the M1 phenotype, enhancing reactivity to microbial components 100, 101. M1 macrophages secrete pro-inflammatory factors (IL-1β, TNF-α, nitric oxide) to mediate anti-infective and anti-tumor immunity, promoting Th1-type immune responses. Surface markers include CD86, CD80, major histocompatibility complex class II (MHC-II), and inducible nitric oxide synthase (iNOS). In contrast to IFN-γ, stimulation with Th2-derived cytokines (IL-4, M-CSF, IL-13) and immune complexes (ICs) converts macrophages to the M2 phenotype 102, inducing distinct surface receptors and effector molecules. M2 macrophages secrete anti-inflammatory cytokines (e.g., IL-10, TGF-β) and chemokines (CCL1, CCL17, CCL18), contributing to tissue repair, immune regulation, and parasite clearance 103. Further differentiation of M2 macrophages yields subpopulations: M2a (anti-inflammatory): CD163, CD206, arginase-1 (ARG-1); M2b (immunomodulatory): CD86, MHC-II 104; M2c (tissue regenerative): CD163, CD206 33; M2d (pro-angiogenic): vascular endothelial growth factor (VEGF), CD14+ 105 (Figure 3).

### Alveolar macrophages

Alveolar macrophages (AMs), as tissue-resident macrophages localized in alveolar tissues, maintain close contact with the external environment. They primarily derive from erythroid-myeloid progenitors (EMPs), with a subset originating from hematopoietic stem cells (HSCs) 106. Trained AMs produce higher levels of IL-6 upon *ex vivo* secondary stimulation with bacteria. Notably, adoptive transfer of trained AMs into naive mice exacerbates bacterial burden and inflammation during subsequent bacterial challenges, whereas protection is achieved only when LPS treatment precedes infection in the same host 107. Binding of GM-CSF to its receptor on AMs induces the master transcription factor PPARγ, upregulating nuclear receptor PPARγ expression to promote catabolism of pulmonary surfactant and maintain its homeostasis 108. Trained AMs exhibit enhanced clearance of heterologous bacteria (*Pseudomonas aeruginosa*) and viruses (respiratory syncytial virus) by secreting IFN-β and pro-inflammatory factors to inhibit pathogen replication 109. Acute influenza A virus (IAV) infection induces TI in mucosal tissue-resident memory AMs, conferring anti-tumor protection against lung metastases. In mouse models, IAV-infected mice show a reduced lung tumor burden and prolonged survival, with this protective effect persisting for 4 months post-infection 110. Dysregulated TI in AMs contributes to persistent inflammation in chronic obstructive pulmonary disease (COPD) 111, while BCG vaccine-induced TI enhances *Mycobacterium tuberculosis* killing in tuberculosis 44. During acute respiratory distress syndrome (ARDS), AM polarization shifts from M1-dominated pro-inflammatory responses in the early phase to M2-mediated tissue repair in the late phase 112. In allergic asthma, AM polarization modulates disease progression, with the inhibition of M2 macrophage polarization shown to ameliorate allergic responses 113.

In summary, AMs play central roles in mucosal immune defense and metabolic remodeling, positioning macrophage-targeted therapies as emerging strategies for treating pulmonary diseases. Elucidating AM biology in basal and diseased states not only deepens our understanding of lung disease mechanisms but also provides critical insights for developing novel therapeutic approaches.

### Splenic macrophages

The spleen, as the largest secondary lymphoid organ in humans, exhibits extensive immune functions in addition to its roles in hematopoiesis and erythrocyte clearance 114. BCG and β-glucan activate the mTOR-HIF1α pathway via NOD2 and Dectin-1 receptors, promoting glycolysis and glutamine catabolism in splenic macrophages. Trained splenic macrophages exhibit enhanced phagocytosis and killing of Salmonella and Candida, accompanied by increased secretion of TNF-α and IL-17 to promote inflammatory responses 115.

In autoimmune diseases like systemic lupus erythematosus, splenic macrophages may remain in an aberrant activation state due to TI, persistently secreting proinflammatory cytokines to sustain autoimmune responses and tissue damage 116. β-glucan-induced splenic macrophages recruit CD8+ T cells to synergistically inhibit tumor growth 117. During sepsis, TI promotes tissue repair via CCR2+ monocyte recruitment, though excessive activation may exacerbate cytokine storms 118. As a reservoir for undifferentiated monocytes and a major site of extramedullary hematopoiesis, the spleen's role in establishing TI was investigated using a β-glucan training model in sham-operated or splenectomized mice. Splenectomy did not modulate pro-inflammatory cytokine production in trained peritoneal cells *in vivo* or eliminate increases in pro-inflammatory circulating monocytes and natural killer cells observed in trained animals. However, splenectomy prevented neutrophilia, a hallmark of TI 119.

Splenic macrophages serve as a systemic immune surveillance and hematopoiesis-regulating “pre-activation hub” for anti-infective immunity. While they play key roles in splenic function regulation, the functional potential of splenic macrophages in TI remains to be fully explored.

### Renal macrophages

Renal macrophages, as tissue-resident immune cells in the kidney, are primarily distributed in glomeruli and the tubulointerstitium, playing critical roles in maintaining renal homeostasis, clearing pathogens and cellular debris, and regulating immune responses 120. The M1/M2 polarization of macrophages is a highly dynamic process involving signaling pathways such as nuclear factor κB (NF-κB), Janus kinase/signal transducer and activator of transcription (JAK/STAT), and phosphatidylinositol 3-kinase (PI3K)/protein kinase B (AKT). NF-κB, by stimulating the release of pro-inflammatory factors like COX-2, iNOS, TNF-α, and IL-6, collaborates with the NLRP3 inflammasome to drive inflammation in diabetic kidney disease (DKD) 121. Epigenetic reprogramming maintains key nodes of the NF-κB pathway (e.g., IκB kinase) in a partially phosphorylated state, enabling rapid signal amplification upon secondary stimulation to shorten response time and enhance cytokine secretion. Cytokines (IL-1β, IL-6, TNF-α), hormones, and growth factors activate JAK-associated kinases, leading to STAT phosphorylation, dimerization, and nuclear translocation to regulate gene transcription in DKD inflammation. Notably, renal STAT-1 phosphorylation is significantly increased in DKD patients, positively correlating with expression of triggering receptor expressed on myeloid cells 1 (TREM-1). Experimental evidence shows that reducing STAT-1 phosphorylation in macrophages decreases TREM-1 expression, promotes M2 polarization, and alleviates high-glucose-mediated inflammation 122. The PI3K/AKT pathway regulates M1/M2 polarization, particularly in M2 macrophage activation 123, 124. In a streptozotocin (STZ)-induced DKD mouse model, inhibiting LINC00323 expression mitigates renal damage. *In vitro*, LINC00323 reduces PI3K and AKT phosphorylation, upregulates M1 marker CD86, increases TNF-α secretion, and decreases IL-10 expression 125. A research team from The Chinese University of Hong Kong discovered that hyperactivated macrophages can transdifferentiate into myofibroblasts to accelerate renal fibrosis, a process driven by aberrant expression of the neuronal gene Pou4f1. Targeting Pou4f1 in macrophages effectively prevents renal fibrosis. In a mouse model of renal fibrosis, Tseng et al. found increased M2a and M2c macrophages, while histone deacetylase inhibitors suppress M2a infiltration, enhance M2c expression, inhibit myofibroblast activation, and reduce fibrosis 126. Pyruvate kinase M2 (PKM2) is upregulated in chronic kidney disease (CKD) mouse kidneys, increasing lactate production. Inhibiting PKM2 alleviates renal fibrosis in unilateral ureteral obstruction (UUO) mice. Mechanistically, lactate induces TGF-β1 expression via histone H3 K18 lactylation in tubular cells, activating Smad3 in M2 macrophages to promote fibrogenic gene expression and initiate macrophage-myofibroblast transition (MMT) 127. Targeting MMT mitigates renal fibrosis-for example, adenosine A2A receptor antagonist MRS1754 reduces renal dysfunction, glomerular fibrosis, and sclerosis in STZ-induced DKD rats by inhibiting MMT 128. α-Naphthoflavone protects against DKD progression by reducing COX-2 production and M1 macrophage infiltration in STZ-induced mice, whereas Wang et al. showed that COX-2 deficiency in hematopoietic cells/macrophages increases M1 infiltration, promotes M2-to-M1 transition, and exacerbates proteinuria and fibrosis, highlighting the need for further studies on COX-2's role in DKD 129.

TI modulation influences kidney transplant survival 130. rBCG-s1PT-immunized SCID mice show reduced renal fungal burden and improved survival after Candida challenge, indicating cross-protection against nonspecific microbes 131. oxLDL-induced TI accelerates renal injury by activating mTORC1 to promote macrophage-myofibroblast transition 132. In mice, PSMP gene deletion or neutralizing antibodies improve acute kidney injury (AKI) from ischemia-reperfusion, rhabdomyolysis, or cisplatin. Mechanistically, PSMP deficiency reduces CCR2+Ly6Chi/F4/80low pro-inflammatory macrophages and suppresses M1 polarization in AKI kidneys 120. A high-salt diet potentiates macrophage TI, driving pro-inflammatory macrophage migration via CCL2-CCR2 signaling and mTORC1 hyperactivation, leading to recurrent renal injury and fibrosis 133.

In summary, renal macrophages are intricately linked to metabolic inflammation and fibrosis, playing pivotal roles in TI. Modulating their polarization and metabolic reprogramming holds promise for controlling kidney disease progression. Future research should dissect the mechanisms of TI and develop targeted therapeutics to provide novel targets for kidney disease intervention.

### Hepatic macrophages

Hepatic macrophages (Kupffer cells), as tissue-resident macrophages in the liver, occupy a unique niche directly exposed to gut microbiota metabolites and pathogen-associated molecular patterns (PAMPs) in portal venous blood, potentially maintaining a state of “low-level training” 134. They play dual roles in TI, serving as both immune sentinels and metabolic regulators.

Exercise-induced HMGB1 release from cardiomyocytes induces TI in hepatic macrophages via the “HMGB1-TLR4-IRG1” axis, promoting itaconate production and Nrf2 signaling activation to protect against hepatic ischemia-reperfusion (I/R) injury 135. Conversely, aberrant TI under metabolic disorders or disease states drives the progression of non-alcoholic fatty liver disease (NAFLD) and cirrhosis. β-glucan stimulation of hepatic macrophages induces squalene epoxidase (SQLE)-catalyzed formation of 24(S),25-epoxycholesterol (24(S),25-EC), which acts as a ligand for liver X receptors (LXRs) to activate LXR/RXR heterodimers and enhance transcription of anti-tumor genes (e.g., IFN-β, IFN-γ) 136.

OxLDL-induced TI potentiates inflammatory responses of hepatic macrophages to secondary stimuli (e.g., TLR2 agonist Pam3cys), sustaining secretion of IL-6, TNFα, and other cytokines to exacerbate vascular inflammation and plaque formation. This effect is enhanced by LXRα activator (e.g., T1317) and attenuated by LXRα inhibitor 63. Hepatic ischemia-reperfusion injury (IRI) and ischemic preconditioning (IPC)/postconditioning (IPO) induce classical (caspase 1) and non-classical (caspase 11) inflammasome regulators to form inflammatory memory. Activation of caspase 1 in IRI, while double knockout of caspase 1 and 11 (Casp DKO) mitigates liver injury by reducing DNA damage and cell death. Such TI exacerbates inflammatory cascades in IRI, promoting hepatocellular dysfunction and tissue damage, with its aberrant activation potentially contributing to other liver diseases like NAFLD 137. Trypanosome infection induces infiltration of monocyte-derived macrophages, which coexist with embryonic-derived Kupffer cells and establish TI via chromatin accessibility changes and upregulation of IFN-γ signaling-related genes. TI enhances hepatic resistance to subsequent bacterial infections, manifesting as improved bacterial clearance and survival 138.

### Intestinal macrophages

The intestine serves as a critical organ for absorbing exogenous nutrients, supplying the body with essential substances through a complex process. Abundant macrophages reside in the intestine, leveraging their heterogeneity to maximize nutrient absorption, maintain microenvironmental homeostasis, and sustain physiological balance 139. Large populations of macrophages inhabit the intestinal lamina propria and Peyer's patches, persistently exposed to commensal microbiota, food antigens, and potential pathogens. Studies show that butyrate, a short-chain fatty acid produced by gut commensals, inhibits histone deacetylase 3 (HDAC3), increasing histone acetylation at antimicrobial peptide and cytokine loci. This drives monocyte-to-macrophage differentiation, reduces mTOR activity, modulates epigenetic modifications, and enhances LC3-associated host defense with antimicrobial peptide production, thereby improving clearance of intestinal pathogens 140. Small-particle (90-45 µm) rice bran-derived arabinoxylans critically induce Dectin-1-dependent epigenetic reprogramming in intestinal macrophages, thereby establishing trained immunity. This fortifies intestinal barrier integrity and prevents pathogen invasion and mitigating inflammation associated with increased intestinal permeability (e.g., IBD). Notably, these particles reverse endotoxin tolerance in macrophages, restoring subsequent LPS challenge 141. Stimulation with Lactobacillus plantarum downregulates NF-κB-p65-regulated pro-inflammatory genes (TNF, IL-1β, IL-6) in intestinal macrophages, reducing pro-cytokine release and promoting anti-inflammatory homeostasis. Concurrently, it suppresses reactienhancen species pathways, decreases glycolytic and respiratory rates, enhances intracellular bacterial survival, and sustains intestinal anti-inflammatory balance 142.

TI in macrophages confers long-term immune memory through metabolic reprogramming (e.g., glycolysis, glutamine catabolism) and epigenetic modifications, exhibiting dual roles in anti-infection, anti-tumor responses, or pro-/anti-inflammatory regulation across different organs. Targeting TI inducers or metabolic/epigenetic pathways has emerged as a potential therapeutic strategy, though balancing protective and pathological effects remains critical (Table 1).

The mechanistic complexity of TI and clinical translation urgently demands interdisciplinary technical integration and precision research. Single-cell RNA sequencing (scRNA-seq) and spatial metabolomics enable tracking of spatiotemporal distribution and functional heterogeneity of macrophage subsets post-TI induction. For instance, influenza virus-infected alveolar macrophages form long-lived M1 subsets with coordinated activation of H3K4me3-modified antiviral genes and glycolytic genes (HK1). Genetic lineage tracing (CRISPR-Cas9 labeling) clarifies intergenerational transmission of epigenetic imprints, dissecting rules of memory maintenance or attrition. Concurrently, multicenter clinical cohorts validate efficacy of TI in infectious diseases and “cold tumors”, defining predictive values of biomarkers. Deciphering dynamics via single-cell multi-omics, expanding therapeutic windows with precision targeting, and optimizing individualized protocols will facilitate the transition of TI from “concept validation” to “precision medicine tool,” offering a novel paradigm for complex disease immunotherapy. Future efforts should dissect regulatory logic across molecular-cell-organismal layers and develop safe, controllable interventions for infections, tumors, and autoimmune diseases. Artificial induction of TI via epigenetic editing (e.g., targeted histone locus modification via CRISPR-Cas9) may emerge as a key direction to break through limitations of traditional vaccine design.

## Research on TI in Macrophages within the Context of Disease

### Cancer

The majority of cancer immunotherapy strategies predominantly revolve around acquired immunity, particularly with tumor-associated T cells. The application of such strategies is severely constrained by the specific type of cancer afflicting a patient as well as the potential for severe adverse side effects. Given that tumor cells have devised a variety of evasion mechanisms to circumvent immune surveillance by antigen-specific T cells, exploiting the antigen-agnostic properties of TI may offer certain advantages 143. Research demonstrated that β-glucan-induced TI exhibits potent anti-improvement efficacy, a process reliant on IFN signaling that operates independently of the host's adaptive immunity 144. In several scenarios, the enhanced immune function elicited through TI holds substantial importance for the investigation of cancers such as bladder, pancreatic, and lung cancer. BCG-trained bone marrow-derived macrophages (BMDMs) inhibit tumor growth via IFN-γ release and reduce pro-tumor inflammatory responses by regulating chemokines (e.g., decreasing MCP-1), representing a novel function of TI 145. At the local level, during BCG infusion in patients with bladder cancer, multiple molecules, including PAMPs and DAMPs, can be detected within the bladder microenvironment, serving as secondary stimuli for BCG-induced cells; From a systemic perspective, repeated BCG infusion therapy instigates a Th1 cell-skewed cytokine response in the urethral epithelium, leading to the recruitment of a significant number of white blood cells to the bladder and an augmentation in cytokine production, thereby conferring a beneficial influence on subsequent anti-tumor immune responses 146. BCG stimulation leads to recruitment of M1 macrophages into the tumor microenvironment (TME), followed by a surge in IFN-γ production. Moreover, the analogous mechanisms observed in BCG-treated bladder cancer seemingly extend to BCG-treated hepatocellular carcinoma (HCC) 77. Clinical trials have also confirmed that TI induced by BCG vaccination alleviates bladder cancer. A research team found that intravesical BCG administration increases production of TNF and IL-1β in patients, effectively inducing TI. This not only reduces respiratory infections in vaccinated individuals but also enhances local anti-tumor immunity 147. Tumor-associated macrophages (TAM) represent one of the most prevalently infiltrated immune cells within the tumor immune microenvironment, and the quantity of TAM in pancreatic cancer correlates with overall survival rates. TAM can foster tumor progression by facilitating angiogenesis. Experimental investigations divulged that β-glucan is transported to the pancreas, prompting monocytes/macrophages reliant on CCR2 influx to exhibit TI signatures. In an in-situ model of pancreatic ductal adenocarcinoma, β-glucan stimulation led to a significant reduction in the tumor burden of cancer - bearing mice. Moreover, compared with the control group, the survival time of these mice was extended, and the survival rate was enhanced. Additionally, when β-glucan stimulation was combined with immunotherapy, this effect was further amplified 148. β-glucan-induced TI can also be integrated with the irreversible electroporation (IRE) technique, substantially reducing local and distant tumor loads in mice harboring *in situ* pancreatic cancer (PC) tumors 149. Experimental studies have identified a previously uncharacterized non-coding RNA species, circITGB6, which is strikingly elevated in both tumor tissues and serum samples of chemoresistant ovarian carcinoma (OC) cases and correlates with adverse clinical outcomes. Elevated levels of circITGB6 promotes M2-polarized macrophage-mediated cisplatin resistance in preclinical models 79. In the context of lung cancer, the application of TI principally aims to attenuate tumor lung metastasis, and whole glucan particle (WGP)-induced TI can effectively govern tumor metastasis. Blocking sphingosine-1-phosphate(S1P) biosynthesis and mitochondrial fission disrupts WGP-induced TI and its inhibitory effect on lung metastasis. WGP can also induce TI in human monocytes and elicit antitumor activity, thereby potentiating antitumor immunity 150.

The innovative potential of TI in cancer immunotherapy lies in its ability to transcend conventional T cell-centric paradigms by utilizing antigen-agnostic stimulants such as β-glucans and BCG to prime myeloid cells, thereby reprogramming the immunosuppressive TME and counteracting immune evasion mechanisms. This approach offers a novel therapeutic paradigm for “immune-cold” tumors characterized by insufficient T cell infiltration. Notably, TI demonstrates cross-cancer applicability: BCG instigates dual antitumor effects in bladder cancer through localized release of PAMPs/ DAMPs and systemic induction of Th1-skewed cytokine cascades, while β-glucans reverse pro-angiogenic functions of TAMs in pancreatic ductal adenocarcinoma via CCR2-dependent monocyte recruitment. Whole glucan particles (WGPs) further suppress lung metastasis by targeting sphingosine-1-phosphate (S1P) biosynthesis and mitochondrial dynamics. Synergistic efficacy is achieved through combinatorial regimens integrating TI inducers with chemotherapy or physical modalities like irreversible electroporation (IRE), which enhances immunogenic cell death. Nevertheless, clinical translation faces challenges including undefined dosing protocols, interpatient heterogeneity in TME composition, paradoxical roles of metabolic targets, and risks of systemic hyperinflammation. Addressing these challenges requires integrating multi-omics approaches with precision medicine strategies to optimize TI-based therapies while mitigating off-target effects.

### Infectious diseases

Infectious diseases are defined as those caused by pathogens (such as viruses, bacteria, fungi, parasites) invading the human body and triggering infectious processes. These pathogens infiltrate the organism through diverse routes, including the respiratory tract, digestive tract, and skin, and subsequently proliferate within susceptible host cells. This proliferation leads to inflammatory responses and tissue damage within the body, thereby giving rise to a series of clinical symptoms 151. Therapeutic modulation of TI can augment the protective immune responses during the acute phase of infection, impeding the progression of infectious diseases. BCG vaccination exhibits the most pronounced impact on respiratory tract infections, especially those caused by viruses 152. Current experiments have verified that BCG-induced TI demonstrates protective effects against infections caused by herpes simplex virus, hepatitis virus, enterovirus, and others. However, the response mechanisms of BCG vaccination to these viral infections vary, warranting further in-depth investigation. Some individuals, even when prolonged contact with patients with infectious tuberculosis, do not progress to active or latent tuberculosis infection (LTBI). This “early clearance” of *Mycobacterium tuberculosis* is linked to with a history of BCG immunization 153. BCG vaccination induces CX3CR1⁺ T cells (adaptive immune component) to secrete IFN-γ, thereby activating persistent antibacterial functions of macrophages and ultimately achieving broad-spectrum protection against heterologous infections 154. Additionally, neonatal BCG vaccination followed by administration of formaldehyde-inactivated respiratory syncytial virus vaccine (FI-RSV+Al (OH)3) promotes trained macrophages to secrete pro-inflammatory cytokines (e.g., IFN-γ, TNF-α) and enhance antigen-presenting functions. This elicits biased induction of Th1-type immune memory, concomitantly inhibiting excessive activation of Th2/Th17 cells to alleviate pulmonary inflammation and mucus secretion 155. MV130, an inactivated multi-bacterial mucosal vaccine, can safeguard patients against recurrent respiratory tract infections. MV130 treatment promotes mTOR phosphorylation to activate HIF-1α, driving glycolysis. Pulmonary alveolar macrophages from MV130-treated mice maintain heightened TI, exhibiting elevated TNF-α levels in lung tissues upon viral reinfection for rapid pathogen clearance 156. Additionally, *Candida albicans* V132 induces TI while concomitantly eliciting enhanced mitochondrial oxidative phosphorylation (OXPHOS), manifested by elevated oxygen consumption rate (OCR) and spare respiratory capacity (SRC). This mechanism potentiates the response of the polybacterial vaccine MV140 against urogenital infections, providing a theoretical basis for developing TI-based combination vaccines 84. Such strategies are particularly suitable for recurrent infection scenarios requiring circumvention of antibiotic resistance. For other antibiotic-resistant Gram-negative bacteria, immunological trained also confers resistance against reinfection 157. TI also manifests a similar protective effect in Candida dubliniensis infections 158. Clinical trials have confirmed that leveraging TI in macrophages reduces parasitic infections. For instance, β-glucan-induced TI stimulates IL-32 expression to enhance anti-infective capacity 159. In BCG-vaccinated individuals, yellow fever virus challenge elicits a blunted viremia peak, which correlates inversely with IL-1β induction levels. BCG vaccination elevates basal levels of pro-inflammatory cytokines (IL-6, TNF-α) without altering anti-inflammatory factors, establishing a “pro-inflammatory bias” that facilitates rapid pathogen clearance 41. Additionally, BCG-vaccinated infants exhibit downregulated anti-inflammatory factors (IL-10, IL-1RA) and chemokines (MCP-1, MIP-1α/β) in responses to TLR2/7/8 ligands and heterologous bacteria/fungi, while maintaining pro-inflammatory cytokine balance. This also demonstrates transgenerational effects of maternal BCG vaccination, which influences infant TI via placental antibody transfer or epigenetic mechanisms 160. In elderly individuals with immunosenescence-characterized by increased monocyte epigenetic heterogeneity-BCG still induces robust TI phenotypes. In high-tuberculosis-burden regions, BCG may concurrently protect against tuberculosis and heterologous infections (bacteria, viruses) by enhancing macrophage pro-inflammatory responses, compensating for limited accessibility to other vaccines 152, 161. In summary, these studies indicate that TI holds potential therapeutic efficacy in a variety of infectious diseases, capable of fortifying the body's defense mechanisms and reducing the incidence of infections. The cross-defense provided by TI exhibits remarkable efficacy in antibacterial and antiviral contexts, furnishing an important reference for the design of vaccines and vaccination schedules.

### Autoimmune diseases

Autoimmune diseases, characterized by immune dysregulation, constitute a group of disorders with highly complex pathogenesis mechanisms, involving aberrant regulation of diverse immune cells and signaling pathways. In recent years, the potential role of TI within this domain has progressively garnered attention. Macrophages derived from patients with autoimmune diseases can modulate cell metabolism and epigenetic modifications via the mTOR signaling pathway, thereby inducing a substantial production of cytokines and manifesting the traits of TI 162. This phenomenon lays a theoretical foundation for subsequent investigations into its functions in specific autoimmune conditions.

Rheumatoid arthritis (RA) is an inflammatory ailment that affects the joints. The autoantibodies in RA, such as anti-citrullinated protein antibody IgG (ACPA IgG), can induce TI in macrophages, accompanied by the secretion of proinflammatory cytokines. This mechanism drives the chronicization of synovial inflammation and tissue damage in RA, providing a theoretical basis for early intervention and targeted metabolic/epigenetic therapies 163. Among them, M1 macrophages assume a central position in promoting inflammation and bone destruction. The hypoxic microenvironment within RA synovium exacerbates M1 metabolic polarization by suppressing mitochondrial oxidative phosphorylation, thereby shifting energy metabolism toward a pro-inflammatory phenotype and disrupting the M1/M2 macrophage dynamic balance. Additionally, TLR2/4 overexpression in RA synovial tissue synergizes with IFN-γ signaling to potentiate M1 polarization, establishing a feedforward loop that sustains chronic tissue inflammation 164.

Systemic lupus erythematosus (SLE) is an immune-mediated disorder capable of assaulting nearly all organs throughout the body. Its cardinal feature lies in the erroneous targeting of self-tissues by the immune system, which drives the secretion of cytokine storm components and immune cell recruitment 165. The early research on SLE mainly focused on some adaptive immune cells. Currently, there are more and more studies on some innate immune cells such as macrophages in SLE 166. Under the stimulation of factors such as IFN-γ, macrophages polarize towards the M1 phenotype and release pro-inflammatory cytokines, influencing the progression of SLE and potentially triggering secondary atherosclerotic lesions. Conversely, when subjected to factors like IL-4, they shift towards M2 macrophages, subsequently affecting SLE and leading to secondary neutrophilic and granulomatous dermatitis 167. It has been reported that in SLE patients, the increased expression of pro-inflammatory factors bears resemblance to the highly expressed cytokines in TI, such as IL-6 and IL-1β. Moreover, myeloid cells and bone marrow progenitor cells in SLE patients exhibit the characteristics of TI, continuously exacerbating the inflammatory state and fueling disease progression 168.

Behçet's disease (BD) is a complex, recurrent inflammatory disorder with both autoinflammatory and autoimmune constituents 169. Macrophages, as pivotal components of innate immunity implicated in BD progression, display an upregulated p-p65 when treated with BD serum. Notably, NF-κB inhibitors can attenuate the M1-like phenotype stimulated by BD serum. Hence, M1 macrophages hold promise as potential therapeutic targets for BD 170. Research has also unveiled that TNF-α, an abnormally elevated cytokine in neutrophils treated with BD serum, can induce the expression of TRPM2 channels, and classic TNF inhibitors can block this effect, effectively alleviating the overactivation of BD neutrophils and vascular inflammation 171. Compared to other diseases, studies on TI in multiple sclerosis (MS) are relatively scant. Nevertheless, the latest research indicates that BCG vaccination can prevent the development of experimental autoimmune encephalomyelitis and concurrently suppress the inflammatory disease activity in MS 172.

TI exhibits a paradoxical role in autoimmune disorders, functioning as a double-edged sword that can amplify inflammatory responses through metabolic-epigenetic reprogramming in conditions such as RA and SLE, while paradoxically being harnessed by inducers like BCG to elicit protective immune modulation in MS. This duality introduces a novel paradigm for understanding autoimmune pathogenesis. However, the cell type-specific regulatory networks governing TI remain incompletely resolved, and current therapeutic interventions lack precision. Future investigations should integrate single-cell multi-omics to delineate the spatiotemporal dynamics of TI within distinct immune cell subsets. Concurrently, developing precision-targeted therapeutic strategies against key epigenetic modifiers may enable the conversion of TI-driven disease exacerbation into clinical benefits, thereby bridging mechanistic insights with translational applications.

### Atherosclerosis

Atherosclerotic cardiovascular disease (ASCVD) is a disease characterized by plaque formation due to persistent subclinical inflammation of the arterial wall 173. The potential association of TI in its pathogenesis has attracted considerable attention. Given that TI can enable cells to exhibit sustained hyperresponsiveness, it is hypothesized to potentially play a crucial role in sustained inflammatory such as ASCVD.

Studies have confirmed that the mechanism of TI can upregulate the expression of a series of pro-atherosclerotic genes, potentially driving the development of atherosclerosis 174. On the one hand, high concentrations of extracellular glucose can promote the expression of inflammatory genes through glycolysis-dependent mechanisms inducing TI, exhibiting pro-atherosclerotic characteristics. What's more, transplanting bone marrow from diabetic mice into normal mice increases the degree of atherosclerosis in the aortic root. TI exhibits remarkable long-term durability, a feature further corroborated by the enrichment of RUNX1 target genes in macrophages within human atherosclerotic plaques-findings that underscore its clinical relevance in human pathobiology 90. On the other hand, it has been found that myocardial infarction (MI) can induce TI, thereby accelerating the atherosclerotic process. In murine models of TI, heightened expression levels of spleen tyrosine kinase (SYK), lysine methyltransferase 5A (KMT5A), and CCHC-type zinc finger nucleic acid-binding protein (CNBP) are observed, using siRNA-mediated knockdown of KMT5A or CNBP to reduce the activity of SYK may slow down the development of atherosclerosis after MI 175. Although it is assumed that TI induces atherosclerosis, there is currently no direct evidence indicating that TI is the root cause of atherosclerosis formation. Many pathways involved in TI can both exert pro-atherosclerotic functions and operate independently of TI, so the actual contribution of TI to atherosclerosis becomes adrift. The example of the NLRP3 inflammasome can be well explained. As a key mediator of Western diet-induced TI, it may promote atherosclerosis, but it can also drive atherosclerosis development in TI-independent manners 70, 176. IFNs act as training stimuli to promote atherogenesis by inducing cellular metabolic reprogramming and proinflammatory cytokine release. Concurrently, TI influences type I IFN production and signaling responses, establishing a positive feedback loop 177. Lipopolysaccharide (LPS)-treated bone marrow-derived macrophages (BMDMs) maintain TLR4 dimers and LR levels-even six days after stimulus removal-with significantly elevated TLR4 density in lipid raft fractions. This “memory” phenotype elicits heightened expression of IL-6, IL-1β, and Cxcl2 in response to the TLR2/1 agonist Pam3CSK4, manifesting as a hyperinflammatory response. Inflammarafts in non-foamy macrophages sustain epigenetic activation of proinflammatory genes (e.g., IL-6, IL-1β) through persistent activation of pathways such as NF-κB, constituting TI. Even after stimulus clearance, this state enables excessive inflammatory responses to secondary challenges, driving atherogenic progression 178.

**Figure 4:**
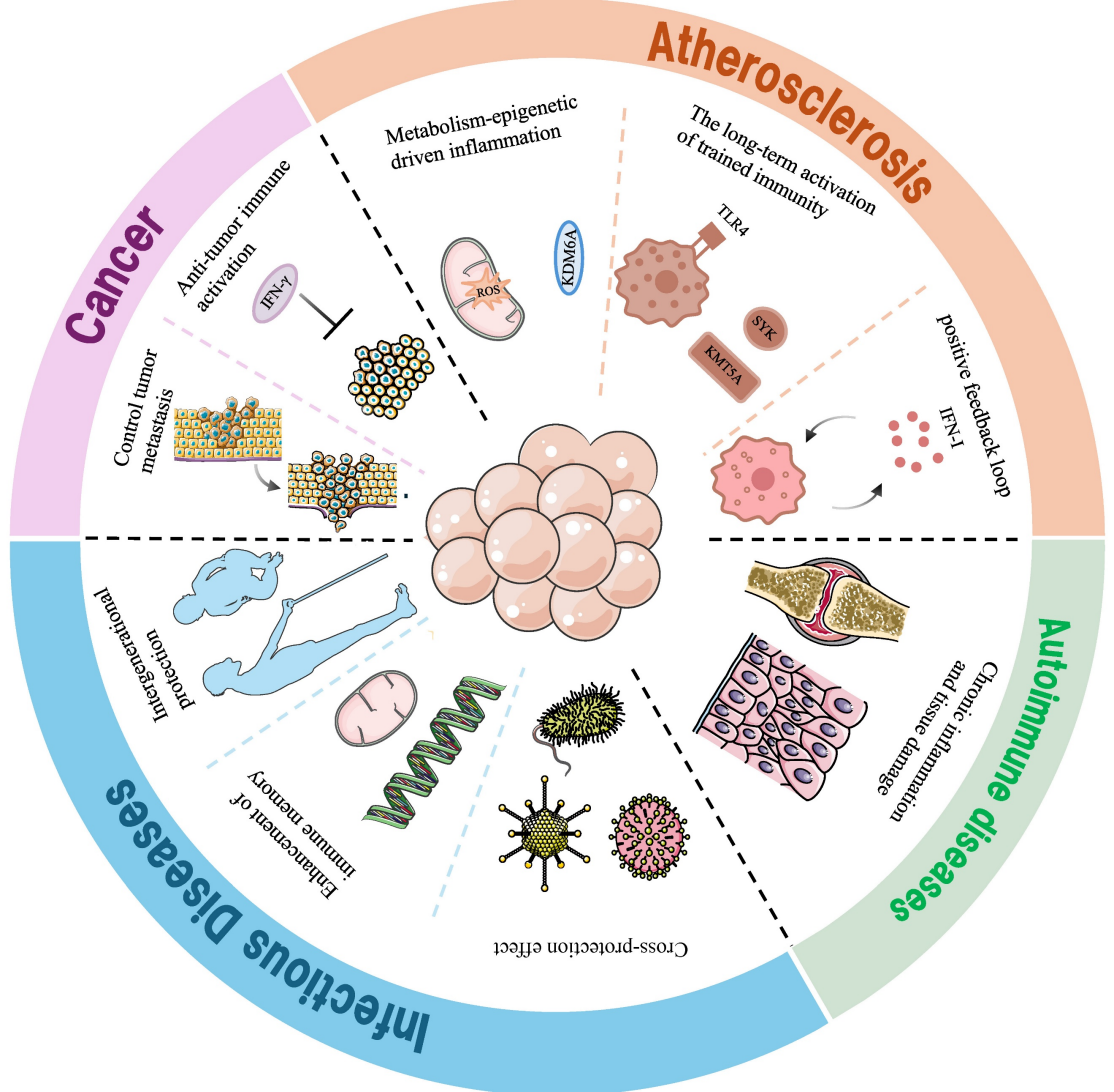
Trained Immunity in Macrophages: Disease-Specific Roles and Mechanism. In Cancer: TI primarily orchestrates anti-tumor immune activation and suppression of metastatic dissemination. TI-reprogrammed macrophages enhance tumoricidal functions and remodel the immunosuppressive tumor microenvironment. In Atherosclerosis: TI drives sustained, maladaptive inflammation via metabolic-epigenetic reprogramming. This establishes a self-perpetuating, pro-atherogenic state characterized by persistent macrophage activation and a pathogenic positive feedback loop accelerating plaque progression. In Autoimmune Diseases: Aberrant TI induction contributes to chronic inflammatory milieus and irreversible tissue damage. TI-primed macrophages perpetuate autoimmune pathology through dysregulated cytokine production and loss of immune tolerance. In Infectious Diseases: TI confers broad-spectrum antimicrobial defense, reinforcing innate immune memory against heterologous pathogens. This enhanced immunological preparedness provides cross-protection and may exhibit transgenerational effects, bolstering host defense across generations. IFN-γ, Interferon-γ; ROS, Reactive oxygen species; KDM6A, Lysine Demethylase 6A; SYK, Spleen tyrosine kinase; KMT5A ,Lysine Methyltransferase 5A.

Due to the exposure of ASCVD patients or those at risk to various complex risk factors throughout their lives, significant heterogeneity exists in the TI induced in patients. Therefore, strategies for treating ASCVD based on TI may need to be individualized, and the characteristics of TI induced by different ASCVD risk factors urgently require in-depth investigation (Figure 4).

## Treatment Prospects and Limitations

TI exerts an effect on infections, malignancies, and autoimmune diseases, suggesting that therapeutic strategies targeting TI may effectively regulate the innate immune response in disease states. Therefore, optimizing TI to enhance disease resistance has become a key objective. However, current treatments targeting TI still face numerous challenges. Nano-immunotherapy and vaccine immunotherapy are relatively mature treatment methods at present.

### Nano-immunotherapy

Nanotechnology is currently developing rapidly and has potential applications in many fields. Nanomaterials can interact with macrophages, making them an ideal platform for regulating TI 179.

Macrophage membrane-coated BCG (M@BCG) constructs form nanoscale biomimetic carriers that selectively target TAMs for endocytic uptake. This biomimetic delivery system elicits TI in TAMs, thereby suppressing Lewis lung carcinoma growth. Notably, the TI induced by M@BCG exhibits long-term durability, as evidenced by sustained antitumor activity 180. Kai Zhou and colleagues developed the M2H@RPK nanotherapeutic system, in which shRNA-LEPR silences the leptin receptor (LEPR) to suppress hyperactivation of the JAK-STAT3 pathway-critical for M1 polarization in TI (TI). Concurrently, the KAFAK peptide directly inhibits NF-κB nuclear translocation, thereby blocking proinflammatory gene transcription. *In vitro* and *in vivo* studies demonstrate that M2H@RPK substantially reduces proinflammatory cytokines, reverses the hyperactivated state of TI by regulating macrophage polarization, controls synovial inflammation, and mitigates joint damage to exert significant therapeutic effects 181. As described above in the context of RA, patients with RA exist in an inflammatory milieu, and the state of TI-itself a proinflammatory state-exacerbates this inflammatory environment. Another type of nanoparticle, namely folate - modified silver nanoparticles (FA-AgNPs), has been experimentally engineered, which actively targets M1-type macrophages. Once these nanoparticles are internalized by cells, they dissolve in response to the elevated levels of intracellular glutathione (GSH), thereby releasing Ag⁺. The released Ag⁺ exerts a series of anti - inflammatory functions. It suppresses anti-apoptotic protein Bcl-2 expression, activates the Caspase-3 pathway, disrupts mitochondrial membrane potential, induces cytochrome c release, and triggers the intrinsic apoptotic pathway to promote M1 macrophage apoptosis-thereby regulating the imbalanced state of TI and contributing to RA therapy 182. This study establishes the first systemic activation of TI via bone marrow-targeted nanotherapeutics, providing a novel paradigm for immunomodulation of the “bone marrow-tumor axis”. Mechanistically, MTP10-HDL nanocarriers activate TI in bone marrow-derived macrophages (BMDMs) through NF-κB and mTOR pathway engagement, exerting dual effects of remodeling TME immune cell composition and upregulating PD-L1 expression. These findings elucidate that nanotherapeutic manipulation of TI at its origin-bone marrow progenitor cells-enables durable activation of macrophage-mediated antitumor functions 183.

Currently, nanomaterial applications primarily aim to suppress the proinflammatory milieu of TI, inducing a phenotypic switch between M1 and M2 macrophages to convert proinflammatory responses into anti-inflammatory cascades for alleviating inflammatory pathologies. Notwithstanding, strategies to precisely manipulate the proinflammatory environment of TI remain to be developed. Exploiting nanotechnology advantages-including precise delivery, nanoscale dimensions, large surface area, and modifiability-along with the identification of additional TI target receptors or key signaling pathways could enable advanced therapeutic strategies for multiple diseases. While leveraging these therapeutic advantages, challenges such as drug resistance, optimal dosage regimen, nanomaterial-induced immune responses, and the impact of diverse training stimuli on nanotherapeutic efficacy must be addressed. Moreover, nanomedicine-based immunotherapy faces substantial translation hurdles: although several candidates have entered clinical trials, few have gained regulatory approval, with most remaining in preclinical stages-a testament to the practical challenges in translating TI-based strategies to the clinic.

### Vaccine immunotherapy

Conventional vaccines are predominantly formulated to evoke adaptive immune memory by precisely targeting pathogens 184. It is now established that the innate immune system can be trained to mount enduring memory responses against both homologous and heterologous secondary challenges. Consequently, TI-based vaccines may be engineered to either directly reprogram TI itself or be co-delivered as integrated platforms to optimize supportive interactions with antigen-specific adaptive immunity-thereby harnessing synergistic adjuvant effects. This concept is exemplified in murine models of tuberculosis therapeutics.

The intranasal engineered BCG vector induces “pulmonary tissue-embedded macrophage populations with recall capacity through the metabolic rewiring through mTOR complex II signaling and hexokinase-1-dependent glycolytic flux modulation and enhances post-TI, providing a new vaccine strategy for combating Mycobacterium tuberculosis infections and neutralizing emergent SARS-CoV-2 variants and targeting the innate immune system through the mucosal surface 185. The mucosal immunotherapeutic agent MV140/V132 coordinates signal transduction through three pivotal pathways in mucosal dendritic cell subsets: the MAP kinase cascade, nuclear factor-κB transcriptional network, and mTOR-dependent metabolic sensors. This tripartite activation axis orchestrates cellular bioenergetic remodeling via mitochondrial dynamics restructuring and chromatin accessibility adjustments through histone modification patterns. The resultant immunometabolic axis stabilization concurrently attenuates microbial resistance evolution, preserves indigenous microflora equilibrium, and disrupts polymicrobial colonization cycles in chronic pelvic mucosal pathologies 186. Rutger J. Röring et al. were the first to discover that MMR vaccination can induce TI processes. Their experimental investigations revealed that following MMR vaccination, long-term alterations occurred in cell transcription and function. Specifically, the secretion of TNF-α and IFN-γ was elevated, and the cell metabolic pathway was upregulated 42. Vaccine immunotherapy enables precise tumor antigen-driven recognition, elicits long-term cellular memory, and exhibits significantly lower side effects than chemotherapy or targeted agents in disease treatment. However, tumor antigen heterogeneity and mutational plasticity limit vaccine-induced immune response rates, while interindividual variability further compromises therapeutic efficacy. Notwithstanding, future research could leverage nanocarriers (e.g., liposomes, exosomes) to encapsulate vaccine antigens-enhancing dendritic cell (DC) uptake efficiency-and co-deliver adjuvants (e.g., CpG) to activate innate immunity. Additionally, vaccine immunotherapy may target epigenetic modifications in TI, such as lactylation and acetylation, through technological innovations to maximize clinical translation.

### Antibody-based intervention therapy

The activation and polarization of macrophages in TI are crucial steps for its function and mediate the secretion of cytokines. Therefore, antibodies against them and their pathways should effectively inhibit or enhance TI.

The specific anti-AXL antibody blocks the M2 polarization of bone-marrow-derived macrophages *in vitro*. Besides tumor cells, targeting AXL in M2-macrophages significantly inhibits the production of CSF-1 and eliminates M2-macrophages in TME, leading to an enhancement of the innate response reflected by M1-like macrophages. Activate physiological TI and restore its anti-tumor inflammatory function. This makes AXL-targeted therapy show potential in non-small-cell lung cancer (NSCLC) and triple-negative breast cancer (TNBC) 187. PY159 is a fucosylated humanized monoclonal antibody directed against TREM1. PY159m, an antibody targeting mouse TREM1. FcγR-mediated TREM1 (triggering receptor expressed on myeloid cells 1) crosslinking activates NF-κB and AP-1 signaling pathways, establishing a “proinflammatory epigenetic memory” in TI. Concurrently, defucosylation of PY159 enhances FcγRIIIa binding affinity, enabling selective elimination of M2-type TAMs in the tumor microenvironment (TME). In syngeneic mouse tumor models, the anti-murine TREM1 antibody PY159m potently augments anti-improvement 188. Biologic agents utilizing immunoglobulin engineering targeting GM-CSF or blocking its cognate receptor interactions have been used in the treatment of rheumatoid arthritis and plaque psoriasis with promising results. Similarly, in clinical trials, neutralizing monoclonal anti-IL-1β antibodies have been validated in the treatment of a variety of inflammatory diseases, such as rheumatoid arthritis, type 2 diabetes, and acute stroke 189.

As described above, antibodies exhibit high specificity for precise targeting of distinct cellular components, minimizing off-target effects. Additionally, their inherent stability and prolonged half-life enable sustained activity to induce TI. However, the potential for allergic reactions to xenogeneic antibodies must be carefully considered. Whereas nanocarriers feature nanoscale dimensions, antibodies-with their large molecular weight-exhibit poor tissue penetrance, resulting in suboptimal drug concentrations at target sites that compromise TI induction efficiency. This may also predispose cytokine storms from excessive TI activation. Collectively, these antibody-based immunotherapies establish a molecular optimization paradigm for TI-targeted antibody drug development. Future advancements must address challenges such as immunogenicity and tissue distribution to maximize therapeutic efficacy.

### Therapy intervening in cell receptor-ligand binding

Intervening in the binding of cell receptors and ligands is one of the therapeutic approaches targeting TI. β-glucan-induced TI depends on the binding of IL-1β to IL -1R. Pharmacological intervention employing IL-1 pathway antagonists (anakinra as IL-1 receptor decoy) and humanized anti-cytokine biologics (canakinumab targeting IL-1β) can inhibit the excessive activation TI of macrophages, reduce joint bone loss 190. There are also studies that have screened the DNA aptamer SL1025 targeting human IL-6. Its PEG aptamer SL1026 inhibits STAT3 phosphorylation in T cells by binding to IL-6 and can be used to treat collagen-induced arthritis 191. JAK-STAT axis serves as a central regulatory node governing macrophage immunometabolic homeostasis. Pharmacological disruption of this pathway using small molecule antagonists exemplified by AG490 has been demonstrated in experimental models to abrogate LPS/IFN-γ-stimulated release of specifically high-mobility group box 1(HMGB1) and interleukin-6 production. Intervening in the JAK2-STAT3 pathway through inhibitors and gene knockout can effectively block NF-κB activation and improve the survival rate of mice. Tocilizumab, an inhibitor of the IL-6 receptor, has been studied for clinical applications, such as playing a role in cardioprotective effects in acute coronary occlusion with ST-segment elevation 192. Preclinical validation studies in models revealed that this TGF-β1-neutralizing aptamer synergistically augmented gefitinib's therapeutic index, mechanistically through dual blockade of Smad2/3 phosphorylation and epithelial-mesenchymal transition suppression, ultimately achieving tumor volume reduction in EGFR-mutant NSCLC xenografts compared to monotherapy 193. A precision-engineered nucleic acid therapeutic targeting tumor necrosis factor-alpha was developed through directed evolution techniques, demonstrating potent neutralization of TNFα-triggered inflammatory cascades. This chemically-modified oligonucleotide conjugate achieved multi-organ protection in preclinical models of cytokine storm syndromes, specifically resolving pulmonary barrier dysfunction and mitigating hepatocyte apoptosis 194. There is also research showing that local application of anti-TNF-α single-stranded DNA aptamer can reduce imiquimod-induced psoriasis inflammation 195. Kulkarni et al. studied aptamer inhibitors against the MCP-1 pathway and found their potential in treating chronic inflammation 196. In addition, a novel integrated strategy for pro-inflammatory factor detection and anti-inflammatory factor treatment was reported: A biosensor based on a structure-switching aptamer can quantitatively and dynamically detect IFN-γ *in vivo* and control the release of aspirin according to the IFN-γ concentration, showing an anti-inflammatory effect 197. A research team developed the second-generation CAR-iMAC (chimeric antigen receptor-engineered innate immune macrophages), which maintains higher and prolonged M1 polarization during antitumor responses, secretes elevated levels of antitumor cytokines, recruits additional NK and T cells, and drives the transition of “cold” solid tumors to “hot” tumors 198 (Table 2).

As discussed, intervening in ligand-receptor binding precisely modulates macrophage responses with high specificity, avoiding systemic immune activation to minimize side effects while inducing more durable TI. However, the human body harbors numerous ligand-receptor pairs, such that intervention in one pathway may disrupt others, leading to off-target effects. Target selection remains challenging, and prolonged intervention may induce receptor downregulation or ligand resistance. A critical challenge for this therapeutic approach is the efficient delivery of intervention agents, with efficacy constrained by the complexity of *in vivo* signaling networks and delivery technology bottlenecks. Future strategies may integrate multi-omics screening for key ligand-receptor pairs, nanocarrier-based delivery systems, and dynamic immune status monitoring. This approach holds potential for infectious diseases and cancer, though further optimization with personalized medicine strategies is required to realize its clinical potential.

In vaccine immunotherapy, both innate immune memory and adaptive immune memory play roles, yet the relationship between innate and adaptive immunity-and the specific contribution of TI therein-remains incompletely elucidated. While TI can be activated across diverse therapeutic approaches, alveolar macrophage-TI requires assistance from adaptive immune T cells, raising questions about the autonomy of TI: to what extent can it control its own activation to avoid excessive inflammatory responses that paradoxically exacerbate disease. In ligand-receptor intervention therapies, emerging stimuli faces the challenge of balancing Dectin1 activation with engagement of other receptors; whether co-activation of distinct receptor classes can potentiate TI remains unaddressed. Current therapeutic strategies, primarily validated in mouse models, confront critical translation hurdles-including species-specific differences between humans and mice, and the impact of sex and hormonal status on TI phenotypes. Moreover, existing approaches often fail to mitigate autoimmune complications or precisely control the magnitude of TI activation. Future research may leverage single-cell sequencing and organoid-based drug screening to refine TI classification-for instance, investigating whether macrophages from different tissues exhibit distinct responses to identical or diverse stimuli. This granular characterization could unlock tissue-specific therapeutic strategies for inflammatory and neoplastic diseases. TI plays a significant role in disease regulation. Although its treatment faces challenges, several therapeutic approaches, including nano-immunotherapy, vaccine-based immunotherapy, antibody-based intervention, and cell ligand-receptor binding intervention, have provided solutions. Nano-immunotherapy, by leveraging the interaction between nanomaterials and macrophages, modulates the immune response to address various diseases. Vaccine-based immunotherapy enhances the body's resistance by TI. Antibody-based intervention targets key aspects of macrophages, while cell ligand-receptor binding intervention blocks relevant signaling pathways. These therapeutic strategies can complement each other, with nanocarriers enabling targeted delivery of ligand-receptor intervention agents to pathological sites-thereby mitigating the onset and progression of related diseases. These therapeutic strategies lay the foundation for regulating TI in disease treatment. However, the underlying mechanisms of some of these approaches remain to be further explored. It is anticipated that these efforts will drive new advancements in clinical treatment.

## Conclusion

TI exerts an impact on the polarization and activation status of macrophages and subsequently influences the onset and progression of diseases within diverse tissues. When exposed to different stimuli, macrophages can activate assorted TI, and this, in turn, affects disease resistance and immune health. As a result, even though TI can shield individuals from infections, it may simultaneously trigger chronic inflammation. TI in tissue-resident macrophages exhibits functional specialization dictated by microenvironmental cues (e.g., metabolic byproducts, pathogen-associated molecular patterns) and epigenetic reprogramming. Alveolar macrophages leverage TI as “metabolic-epigenetic sentinels” for mucosal immunity, splenic macrophages act as “hub regulators” of systemic immunity, and renal macrophages function as “fibrotic drivers” in metabolic diseases. Future investigations should dissect the tissue-specific regulatory networks of TI to enable precision-targeted therapeutics. Studies on related ailments of the alveoli, spleen, and kidneys will enable us to comprehend the mechanisms where macrophages come into play. Given the prominent role of macrophages, they are gradually turning into an alluring target for treating a wide array of cancers and immune conditions. Nonetheless, additional research is still essential to uncover their underlying benefits and potential risks. What's more, in the future, specific elements of the immune mechanisms related to TI, such as epigenetic and metabolic checkpoints, might potentially be employed to formulate targeted interventions for diseases.

## Figures and Tables

**Table 1:** Function of macrophages in different tissues

Organ	Disease	Function	Reference
**Lung**	Viral infections (influenza, RSV)	Maintain OXPHOS metabolism, secrete IFN-β and pro-inflammatory factors to inhibit pathogen replication, and provide long-term antiviral protection (lasting for 4 months)	110
	Tuberculosis	The BCG vaccine for tuberculosis induces TI, enhancing the killing ability against Mycobacterium tuberculosis.	44
	COPD	TI dysregulation leads to persistent inflammation and aggravates airway damage.	111
	Lung tumor metastasis	Influenza virus infection induces TI, forming a tissue-resident memory phenotype and inhibiting lung tumor metastasis.	150
	ARDS	Dynamic changes in polarization status: in the early stage, it is mainly characterized by M1-type pro-inflammatory responses, and in the late stage, it shifts to M2-type mediating tissue repair	112
	Allergic asthma	Inhibiting M2 polarization can improve allergic reactions.	113
**Spleen**	Bacterial/fungal infections (Salmonella, Candida)	BCG/β -glucan activates the mTOR-HIF1α pathway, enhances phagocytic and cytotoxic functions, and promotes the secretion of TNF-α/IL-17.	115
	SLE	TI leads to abnormal activation of macrophages, continuous secretion of pro-inflammatory factors, exacerbating autoimmune responses and tissue damage.	116
	Anti-tumor immune	β -glucan-induced splenic macrophages recruit CD8⁺T cells to synergistically inhibit tumor growth.	117
	Sepsis	Recruits CCR2⁺ monocytes to promote tissue repair, but excessive activation may exacerbate the cytokine storm.	118
**Renal**	DKD	Promotes M1 polarization through the NF-κB, JAK/STAT and PI3K/AKT pathways, and secretes TNF-α/IL-6 to aggravate inflammation and fibrosis.	121 122 125
	Renal fibrosis	The high glucose environment in renal fibrosis induces macrophage-myofibroblast transdifferentiation (MMT), and the TGF-β1-Smad3 pathway drives fibrosis. Histone deacetylase inhibitors can inhibit the infiltration of M2a macrophages.	126 128
	AKI	The deletion of the PSMP gene or neutralizing antibodies reduces the infiltration of CCR2⁺Ly6C⁺ pro-inflammatory macrophages, thereby alleviating the injury.	120
	Kidney transplantation	The serum after kidney transplantation can inhibit trained immunity and increase the survival rate of kidney transplantation	130
	High-salt diet-related renal injury	Enhances TI, drives macrophage migration through CCL2-CCR2 signaling and excessive activation of mTORC1, and accelerates the recurrence and fibrosis of renal injury.	133
**Liver**	IRI	Exercise-induced release of HMGB1 by cardiomyocytes triggers TI through the “HMGB1-TLR4-IRG1” axis, promoting itaconic acid production and Nrf2 pathway activation, thereby alleviating the injury.	135
	NAFLD	Abnormal activation of TI drives the progression of steatosis and liver cirrhosis.	137
	HCC	β -glucan induces squalene cyclooxygenase (SQLE) to catalyze the generation of 24(S), 25-epoxycholesterol, activating the LXR/RXR heterodimer to enhance the transcription of anti-tumor genes (such as IFN-β/γ).	136
	Bacterial infections (such as Pseudomonas aeruginosa)	Trypanosoma infection induces mononuclear macrophages to establish TI, enhancing the liver's ability to resist subsequent infections.	138
**Intestinal**	Intestinal pathogen infection (Salmonella)	Butyrate, a metabolic product of symbiotic bacteria, inhibits HDAC3, enhances the expression of antimicrobial peptides and autophagy-related host defense, and promotes pathogen clearance.	140
	IBD	fortifies intestinal barrier integrity and prevents pathogen invasion and mitigating inflammation associated with increased intestinal permeability	141
	Intestinal immune balance	sustains intestinal anti-inflammatory balance	142

COPD: chronic obstructive pulmonary disease; ARDS: acute respiratory distress syndrome; SLE: Systemic lupus erythematosus; DKD: Diabetic nephropathy; AKI: Acute kidney injury; IRI: Ischemia-reperfusion injury; NAFLD: non-alcoholic fatty liver disease; HCC: Hepatocellular carcinoma; IBD: Components of symbiotic bacteria in inflammatory bowel disease.

**Table 2:** Therapeutic targets for trained immunity with macrophages in diseases

Target Category	Specific Target	Therapeutic Approach	Associated Diseases	References
Metabolic Pathways	mTOR-HIF1α-glycolysis axis	mTOR inhibitors (rapamycin); HIF1α stabilizers	Cancer, Infections	85, 132,133,156
	OXPHOS	PPARγ agonists (pioglitazone)	Autoimmune Diseases	70, 87
	Glycolysis		Infectious Diseases	75,84
Epigenetic Modifiers	H3K4me3 writers/erasers	KDM5 inhibitors	Infections, Autoimmunity	78,79,83,87,90
	Histone lactylation	PKM2 inhibitors	Renal fibrosis	127
Cytokine Signaling	IL-1β/IL-1R	Anakinra, Canakinumab Blocks	RA, type 2 diabetes, and acute stroke	189,190
	IL-6	DNA aptamer	Arthritis	191
	TNF-α	Anti-TNF-α single-stranded DNA aptamer; TNF inhibitors	Psoriasis inflammation, vascular inflammation	171,195
	JAK-STAT	Tofacitinib; AG490	SLE, DKD	88,181,192
	IFN-γ		Inflammation	197
	MCP-1	Aptamer inhibitors	Chronic inflammation	196
Surface Receptors	TLR4	Agonist Pam3CSK4	Atherogenic	178
	TREM1	Anti-TREM1 antibody (PY159)	Cancer	188
	Dectin-1 (β-glucan receptor)	β-glucan nanoparticles	IBD	141
Cellular Reprogramming	M2-to-M1 repolarization	Folate-modified AgNPs	RA	164,182
	Macrophage-myofibroblast transition	A2A receptor antagonist (MRS1754)	Renal fibrosis	127,128,132

mTOR: Mammalian target of rapamycin; HIF1α: Hypoxia-inducible factor -alpha; H3K4me3: Methylation of histone H3 lysine 4; H3K27ac: Acetylation of histone H3 lysine 27; IL-1β: Interleukin-1β; IL-1R: Interleukin-1receptor; IL-6: Interleukin-6; TNF-α: tumor necrosis factor-α; JAK-STAT: Janus Kinase-Signal Transducer and Activator of Transcription; IFN-γ: Interferon-γ; MCP-1: Monocyte chemoattractant protein-1; TLR4: Toll-like receptor 4; TREM1: Triggering receptor expressed on myeloid cells-1; M2:macrophage 2; M1:macrophage 1; PPARγ: Peroxisome proliferator-activated receptor gamma; KDM5: Lysine Demethylase 5B; EZH2: Enhancer of zeste homolog 2.
